# Genetic influence of PPAR-γ rs1801282 and MTRR rs162036 variants on non-small cell lung cancer risk in Egyptians

**DOI:** 10.1007/s12672-026-04972-8

**Published:** 2026-05-07

**Authors:** Yomna F. Metwally, Rasha F. Zahran, Rana R. El Sadda, Sherif Refaat, Afaf M. Elsaid

**Affiliations:** 1 Chemistry Department, Faculty of Science, Damietta University, Damietta, Egypt; 2 Oncology Department, Oncology Center, Mansoura University, Mansoura, Egypt; 3 Genetics Unit, Children Hospital, Mansoura University, Mansoura, Egypt

**Keywords:** Lung cancer, Gene polymorphisms, PPAR-γ, *MTRR*, Egypt

## Abstract

**Background:**

Lung cancer remains the leading cause of cancer-related deaths worldwide. Despite advances in diagnostics and treatment, most patients are diagnosed at advanced stages. Genetic variants such as single nucleotide polymorphisms (SNPs) may improve early detection and risk prediction. This study investigated the association of PPAR-γ rs1801282 and MTRR rs162036 polymorphisms with non-small cell lung cancer (NSCLC) susceptibility in Egyptians and further evaluated their role through a meta-analysis.

**Methods:**

A case–control study was conducted including 127 NSCLC patients and 138 age- and sex-matched healthy controls. Genotyping of PPAR-γ rs1801282 and MTRR rs162036 variants was performed using the T-ARMS-PCR method. Logistic regression, stratified and multivariate analyses, and bioinformatics approaches were applied to assess genetic associations. In addition, a meta-analysis of previously published studies was performed to evaluate the overall association of these polymorphisms with cancer risk.

**Results:**

The PPAR-γ rs1801282 C>G variant showed a significant association with NSCLC risk in both the dominant (adjusted OR = 6.74, *p* < 0.001) and allelic models (adjusted OR = 6.10, *p* = 0.001). Stratified analysis indicated higher susceptibility among males, older individuals, and nonsmokers. No significant association was observed for the MTRR rs162036 variant (*p* > 0.05). The meta-analysis findings were consistent with the overall lack of association between MTRR rs162036 and cancer risk, while supporting a potential role for PPAR-γ rs1801282 in cancer susceptibility.

**Conclusions:**

The PPAR-γ rs1801282 polymorphism may serve as a potential genetic marker for NSCLC risk in Egyptians, whereas MTRR rs162036 shows no significant impact. The combined evidence from the case–control study and meta-analysis highlights the importance of population-specific genetic investigations in NSCLC.

**Supplementary Information:**

The online version contains supplementary material available at 10.1007/s12672-026-04972-8.

## Background

Lung cancer (LC) represents the top cancer in terms of occurrence, invasiveness, and metastasis worldwide. In 2023, lung cancer recorded 127,070 deaths (20.8% of total cancers) and over 238,340 novel cases (12.2% of all cancers) [1]. It is the prime cause of cancer death for men and women, as well as the second most diagnosed cancer, following prostate cancer in men and breast cancer in women [2]. LC affects males approximately two times as often as females [3]. Interestingly, LC rates continue to grow in Egypt, where it is the fourth cancer in terms of mortality and the fifth cancer in incidence [4].

In Egypt, lung cancer ranks among the top leading cancers, placing third in incidence for males and contributing significantly to the national burden with approximately 26,845 new cases reported in 2022. It is the most lethal malignancy overall, with rising annual cases from 16,596 in 2008 to 29,576 in 2020, and an age-adjusted mortality rate of 8.02 per 100,000 population. In North African Arab countries, Egypt accounts for 20.7% of lung cancer deaths, underscoring its high regional mortality ranking [5].

LC is identified as a heterogeneous disease originating from different sites in the bronchi Non-small cell lung cancer (NSCLC) is sorted as the most frequent type (85%) of LC and includes different subtypes: adenocarcinoma (AC), squamous cell carcinoma (SCC), and large cell carcinoma (LCC) [6].

LC is initially asymptomatic; however, diverse signs and symptoms related to its origin and spread typically appear within the advanced phase. Cough is the primary symptom present in 50% to 75% of lung cancer patients. Other symptoms can include dyspnea, chest pain, and hemoptysis [7]. Despite advancements in diagnosis, staging, and treatment strategies, the majority of NSCLC patients (70%) are not diagnosed until they progress to late stages (IIIB–IV) [8]. As a result, the survival rate of NSCLC remains very poor and does not exceed 15% for 5 years in most countries [6].

Although the definite causes underlying LC have been ambiguous, environmental, occupational, and genetic factors are the crucial risk factors for its occurrence. Smoking is the most predominant risk factor, representing more than 80% of LC cases. However, since only 15% of smokers are diagnosed with LC patients, genetic factors are considered the determinant of interindividual differences in LC development [9]. Accordingly, many single nucleotide polymorphisms (SNPs) have been established as candidate biomarkers for developing LC [10].

The methionine synthase reductase (MTRR) is a necessary enzyme for maintaining the remethylation of homocysteine to methionine, a middling step between the folate and methionine cycles [11]. Methionine is formed from 5-methyl tetrahydrofolate, which donates a methyl group to homocysteine, by the methionine synthase (MTR) enzyme and the cofactor cobalamin. Methionine is essential in one-carbon metabolism, where it is converted into S-adenosylmethionine (SAM) compound, a general methyl donor required for several cellular processes such as DNA and histone methylation and molecular biosynthesis [12].

Moreover, the MTRR enzyme acts as an activator of the cofactor cobalamin when it is in its oxidized form, so that it is responsible for the regeneration of the MTR enzyme [13, 14]. It has been found that the lower the function of MTRR, the greater the disruption of the folate cycle and the higher the homocysteine levels [15]. Decreased MTRR activity has also been related to cancer development, which may be due to lower DNA methylation, improper thymidine production, increased uracil intervention into DNA, DNA strand distortion, and lowered nucleotide excision repair efficiency [16].

The *MTRR* gene, located on chromosome 5 (from 5p15.2 to 15.3) in the short arm, is translated into the MTRR enzyme [15, 17]. Different genetic polymorphisms of *MTRR* can greatly affect its activity, thereby modulating DNA methylation, repair, and synthesis [18, 19]. The rs162036 *MTRR* genetic variant involves a nucleotide change from A to G (c.1049A > G) and a switch of lysine with arginine (*p*. Lys350Arg) in the protein. It has been reported that the rs162036 G allele has a positive correlation with nonobstructive azoospermia occurrence [18]. Additionally, it has been revealed that the *MTRR* rs162036 may have a protective role against breast cancer in the Chinese population [20]. However, the impact of this variant on the development of NSCLC has not yet been addressed.

On the other hand, peroxisome proliferator-activated receptor gamma (PPAR-γ) is a crucial transcription factor that constitutes two isoforms, PPAR-γ1 and PPAR-γ2, generated by alternative splicing. PPAR-γ2 is characterized by an extra 30 N-terminal amino acids (AAs), which are responsible for ligand-independent activation and are 5–10-times more efficacious than PPAR-γ [21].

As one of the nuclear hormone receptor superfamily, PPAR-γ is a ligand-dependent transcriptional regulator of genes related to carbohydrate and lipid metabolism, as well as differentiation [22]. Recently, PPAR-γ has garnered considerable interest as an important modulator in carcinogenesis. It has been proposed that PPAR-γ can inhibit tumor growth through the induction of differentiation, apoptosis, and antiproliferation [23].

The *PPAR-γ* gene is found on chromosome 3 (3p25) and is translated into the PPAR-γ protein, which consists of 505 amino acids [24]. The most common polymorphism of the *PPAR-γ* gene is rs1801282 C>G, also known as Pro12Ala. This polymorphism has a substantial impact on PPAR-γ protein structure, dampening its function [25]. Several lines of evidence have established the role of the rs1801282 polymorphism in the development of numerous cancers [26–28]. Nevertheless, limited surveys have concerned the relation between *PPAR-γ* rs1801282 and NSCLC risk. Recent genome-wide association studies (GWAS) and large-scale meta-analyses have substantially advanced the understanding of the genetic architecture of non-small cell lung cancer (NSCLC). Multiple susceptibility loci have been identified across diverse populations, including variants near TERT, TP63, CHRNA5–CHRNA3–CHRNB4, EGFR, and BAT3–MSH5, highlighting the polygenic nature of NSCLC risk. These studies emphasize the involvement of pathways related to telomere maintenance, DNA repair, inflammation, xenobiotic metabolism, and cell-cycle regulation. However, most GWAS have been conducted in European and East Asian populations, with limited representation of Middle Eastern and North African populations. Consequently, population-specific genetic risk factors may remain undiscovered, underscoring the continued importance of candidate-gene association studies in underrepresented ethnic groups, such as Egyptians [29, 30].

Therefore, the first objective of our case–control design was to discover the possible correlation between genetic variations in *MTRR* and *PPAR-γ* and the risk of NSCLC in the Egyptian population. Secondly, all laboratory and clinical parameters of NSCLC patients would be tested relative to the candidate gene variants.

## Subjects and methods

### Study populations

A total of 265 similar ethnic individuals were allocated in this case–control study, comprising 138 hospital-based healthy volunteers and 127 NSCLC patients with primary diagnoses during the same period (2/2023 till 2/2024). Patients who met the following criteria were included in the study: age over 18 years, a confirmed histological or cytological diagnosis of NSCLC (grades I–III), adequate organ function, computed tomography scan results (stages I–IV), no previous cancer treatment, and easily accessible clinical records. NSCLC was staged per AJCC 8th edition (stages I–IV) and graded histologically (G1–G3) well to poorly differentiated, excluding undifferentiated tumors).e Patients with other malignancies, bronchitis, autoimmune disorders, open wounds, asthma, lung abscesses, pneumonia, or tuberculosis (TB) were ruled out from the study. NSCLC cases were classified according to clinical stage I–IV using AJCC/TNM criteria based on imaging and histopathological confirmation. Histological differentiation (grade) was recorded separately but not used for inclusion/exclusion criteria. For the control group, individuals with a family history of cancer or pulmonary diseases were avoided. Chest imaging was done to exclude lung cancer in addition to exclusion of respiratory symptoms or pulmonary disease.

The power calculation was performed using the sample size software G*Power (Version 3.1.9.7), with a significance level (α) of 0.05 and an effect size of 0.24, resulting in a test power (1 − β) of 96.5% [31].

### Data collection

Our team obtained eligible consent from the Medical Ethical Committee, and written agreement forms were assembled from all participants. Clinical information was gathered from the patients' medical records, including their age, gender, smoking status (smoker or non-smoker), surgical history, family history of lung cancer, medical history, radiological checks, histopathologic information (tumor histology and stage), epidermal growth factor receptor (EGFR) gene status, and carcinoembryonic antigen (CEA) levels. The NSCLC classification was accomplished contingent with the criteria of the American Joint Committee on Cancer (AJCC) staging approach [32].

### DNA extraction and genotyping

All study individuals donated 3 ml of EDTA blood for DNA extraction. The GeneJET DNA Purification Kit (Thermo Fisher Scientific, K0781, Lithuania) was used following all manufacturer’s steps. The DNA quantity and purity were measured using the NanoDrop™ 1000 Spectrophotometer and then kept at − 80 degrees Celsius for further testing [33].

The two SNP loci in the *MTRR* (rs162036) and *PPAR-γ* (rs1801282) genes met the criteria of having a minor allele frequency (MAF) > 0.05 in the global population, according to the 1,000 Genome Project (http://www.internationalgenome.org/). Genotyping of *MTRR* and *PPAR-γ* was performed based on the amplification refractory mutation system polymerase chain reaction (ARMS-PCR) approach, which included four primers: two outer and two inner (allele-specific) primers [34]. The primer base pair sequencing for *MTRR* (rs162036) and *PPAR-γ* (rs1801282) (Willofort, UK) is illustrated in Table 1.

**Table 1:** The PCR primer sequences and thermal cycling condition for *MTRR* and *PPAR-γ* genes

SNP	Coding impact	Accession number ^a^	Primer sequences	Product size	PCR conditions
MTRR (rs162036)	Missense variant	**NM_002454.3:c.1049A > G**	FO: 5ʹ-CAGCGTGATCTGCCCTAACAGTGATTCT-3ʹ RO: 5ʹ-TACCAATACCAGCGTATGCCTGTGTTCC-3ʹ FI: 5ʹ-CGTCCTTTTGAAAATAAAGGCAGACACCAA-3ʹ RI: 5ʹ-AGCATCAGGGCTGTTACCTTTCTGCC-3ʹ	G allele: 204 bp A allele: 136 bp	Initial denaturation cycle at 95 °C for 5 min 35 thermal cycles of denaturation at 95 °C for 30 S, annealing at 58 °C for 30 S, and extension at 72 °C for 30 S Final extension cycle at 72 °C for 10 min
PPAR-γ (rs1801282)	Missense variant	**NM_138711.6:c.-8-28078C>G**	FO: 5ʹ-AACTTTTTGTCACAGCTGGCTCCTAATA-3ʹ RO: 5ʹ-CAACGAGCTAAGCATTAAAATACTGGA-3ʹ FI: 5ʹ-GAAACTCTGGGAGATTCTCCTATTGTCC-3ʹ RI: 5ʹ-GTATCAGTGAAGGAATCGCTTTCAGC-3ʹ	C allele: 219 bp G allele: 288 bp	Initial denaturation cycle at 95 °C for 5 min 35 thermal cycles of denaturation at 95 °C for 1 min, annealing at 63 °C for 1 min, and extension at 72 °C for 1 min Final extension cycle at 72 °C for 7 min

^a^ The MTRR variant dataset is available from ClinVAR database (https://www.ncbi.nlm.nih.gov/clinvar/variation/138291/?oq=((141994[AlleleID]))&m=NM_002454.3(MTRR):c.1049A%3EG%20(p.Lys350Arg))

The PPAR-γ variant dataset is available from ClinVAR database (https://www.ncbi.nlm.nih.gov/clinvar/variation/130019/?oq=((135465[AlleleID]))&m=NM_138711.6(PPARG):c.-8-28078C%3EG)

The MTRR assay lacks the reverse internal primer, whereas PPAR-γ includes two internal primers, reflecting the specific design required to discriminate alleles for each SNP. Bold font denotes the position of the variant at the DNA (c.) and protein (p.) levels

The PCR cycling of both *MTRR* (rs162036) and *PPAR-γ* (rs1801282) was carried out by the thermal cycler SimpliAmp™ (Applied Biosystems, USA) according to Table 1. A 2.5% agarose gel electrophoresis was utilized to run the PCR products, followed by ethidium bromide for visualization. The *MTRR* (rs162036) PCR results showed 204 bp for the G-allele and 136 bp for the A-allele (Fig. S1). The *PPAR-γ* (rs1801282) results were obtained at 219 bp with the C-allele and at 288 bp with the G-allele (Fig. S2). Up to 10% of the samples were randomly chosen to confirm the results of PCR, and both sets of results were consistent. The *MTRR* gene is located on the forward (sense) strand of chromosome 5p15.31, indicating that this strand serves as the template for transcription, while the complementary strand is not used for this gene’s mRNA synthesis. The T-ARMS-PCR technique uses two outer primers (FO and RO) and two inner allele-specific primers (FI and RI) to detect each SNP. Differences in the number of functional primers between MTRR (rs162036) and PPAR-γ (rs1801282) reflect sequence-specific optimization needed for efficient amplification and accurate allele discrimination.

### Inclusion and exclusion criteria for meta-analysis

The inclusion criteria for our met-analysis were the following: case-controlled studies or cohort studies, studies investigating the relation between *PPAR-γ* or *MTRR* gene variants and cancer, and studies providing sufficient data on frequencies of genotypes and alleles in order to get relative odds ratios (ORs) and 95% confidence intervals (CIs). Whereas studies available only as abstracts or review articles were excluded.

### Statistics

Statistical tests were computed using IBM SPSS program (version 26.0). The quantitative data were figured as means and standard errors (M ± SE) using the student’s t*-*test. The qualitative data were recorded as numbers and percentages (N%) and analyzed by Fisher's exact test. Hardy–Weinberg equilibrium (HWE) was tested using the Chi-square test to compare the observed and predicted genotypic frequencies within the case and control groups. Allelic and genotypic counts for *MTRR* and *PPAR*-*γ* variants were calculated using Fisher's exact test.

The risk estimation of NSCLC was performed using logistic regression analysis under both genotypic and allelic models [35]. ORs and CIs were determined for all genotypic variants, adjusted for age, gender, smoking, and family history. Stratification analysis by age, gender, smoking, and pathological types was also accomplished using logistic regression. Post-hoc power analysis was performed based on observed effect sizes, allele frequencies, and the total sample size (n = 265) to aid interpretation of non-significant findings.

Moreover, multivariate investigations, including principal component analysis and correlation matrix, were conducted using R programming language software (version 4.4.1) and R Studio (version 2024.04.2 Build 764). The meta-analysis results were obtained using Stata Statistical Software, Release #17 (StataCorp. 2021, College Station, TX: LLC). A two-sided *P* value of less than 0.05 showed a significant statistical test.

Given the modest sample size (n = 265), a post-hoc power calculation was performed. For PPAR-γ rs1801282, the study achieved ~ 80% power to detect an odds ratio ≥ 6.0 at α = 0.05. For MTRR rs162036, power was limited due to the low minor allele frequency (~ 10–15%), which may contribute to the null association observed.

This study was conducted and reported in accordance with the STROBE and STREGA guidelines for observational genetic association studies.

## Results

### Characteristics of the study subjects

All clinical and laboratory features of the study groups were represented in Table 2. The study was categorized into 138 healthy volunteers (average age: 53.4 ± 0.88 years) and 127 NSCLC patients (average age: 55.8 ± 1.03 years). The ratio of males vs. females in the NSCLC group (59.8% vs. 40.2%) was identical to the control group (66.7% vs. 33.3%). Nevertheless, there was a highly significant difference (*p* < 0.001) in smoking status between NSCLC patients (51.2%) and controls (23.9%). Family history accounted for 14 (11%) of the NSCLC patients, whereas it was missing in controls, with statistically significant difference (*p* < 0.001). Histopathological parameters of the NSCLC group showed that 78% had AC, and 96% were in the late stages (III and IV). Clinically, significant differences were found between NSCLC cases and controls in INR, PT, AST, ALT, and total bilirubin (*p* < 0.05). Also, hematological data such as hemoglobin, WBCs, platelets, neutrophils, and lymphocytes showed a significant association different between the two groups (*p* < 0.05).

**Table 2:** Characteristics of the investigated study groups

Parameter	Cases (n = 127)	Control (n = 138)	*P*
*1. Clinical parameters*			
Age, years, M ± SE	55.8 ± 1.03	53.4 ± 0.88	0.074^a^
Age groups, years, (< 55/ > = 55), n (%)	58 (45.7)/ 69 (54.3)	74 (53.6)/ 64 (46.4)	0.22^b^
Sex			
Female/Male, n (%)	51 (40.2)/76 (59.8)	46 (33.3)/92 (66.7)	0.25^b^
Smoking			
Smokers/Nonsmokers, n (%)	65 (51.2)/62 (48.8)	33 (23.9)/105 (76.1)	** < 0.001**^b^
Symptoms			
Positive Cough, n (%)	49 (38.6)	–	NA
Positive Dyspnea, n (%)	44 (34.6)	–	NA
Positive Chest pain, n (%)	40 (31.5)	–	NA
Positive Hemoptysis, n (%)	9 (7.1)	–	NA
Positive family history, n (%)	14 (11)	0 (0)/138 (100)	** < 0.001**^b^
Positive surgical history, n (%)	45 (35.4)	36 (26.1)/ 102 (73.9)	0.099^b^
Positive medical history, n (%)	66 (52)	57 (41.3)/81 (58.7)	0.082^b^
Tumor size (T)			
T1 + T2, n (%)	12 (9.5)	–	NA
T3 + T4, n (%)	115 (90.6)	–	
Lymph node (N)			
N0 + N1, n (%)	31 (24.4)	–	NA
N2 + N3, n (%)	96 (75.6)	–	
Stage			
Stage 1 + 2, n (%)	5 (4)	–	NA
Stage 3 + 4, n (%)	122 (96)	–	
Grade			
Mild, n (%)	3 (2.4)	–	NA
Moderate + High, n (%)	124 (97.6)	–	
Histological types			
Adenocarcinoma, n (%)	99 (78)	–	NA
Squamous cell carcinoma, n (%)	13 (10.2)	–	
Large cell carcinoma, n (%)	15 (11.8)	–	
*2. Biochemical parameters*			
PT (sec), M ± SE	13.11 ± 0.12	12.63 ± 0.07	**0.001**^a^
INR, M ± SE	1.09 ± 0.01	1.05 ± 0.0	**0.002**^a^
ALT (U/L), M ± SE	23.8 ± 1.2	20.4 ± 0.61	**0.014**^a^
AST (U/L), M ± SE	24.4 ± 1.4	19.0 ± 0.52	** < 0.001**^a^
Total bilirubin (mg/dL), M ± SE	0.56 ± 0.02	0.65 ± 0.01	**0.001**^a^
Creatinine (mg/dL), M ± SE	0.87 ± 0.01	0.85 ± 0.02	0.493^a^
Hemoglobin (g/dL), M ± SE	12.8 ± 0.16	12.36 ± 0.14	**0.034**^a^
WBCs count (× 10^9^/L), M ± SE	10.4 ± 0.44	8.52 ± 0.16	** < 0.001**^a^
Platelet count (× 10^9^/L), M ± SE	305 ± 8.7	255 ± 7.3	** < 0.001**^a^
NEUT (%), M ± SE	66.2 ± 1.04	58.6 ± 1.09	** < 0.001**^a^
LYMPH (%), M ± SE	22.6 ± 0.93	33.0 ± 1.03	** < 0.001**^a^
*3. Tumor markers and genetic markers*			
CEA (ng/mL), M ± SE	35.47 ± 6.26	–	NA
CEA, (Positive/Negative), n (%)	80 (63)/47 (37)	–	
EGFR (Mutant), n (%)	31 (24.2)	–	NA

^a^Data are presented as mean and standard error, *t* test was applied, ^b^Data are presented as numbers with percentages, Fisher’s exact test was applied; bold values signify *p* < 0.05.*SE* standard error, *CEA* carcinoembryonic antigen, *EGFR* epidermal growth factor receptor, *NA* not applicable

**Fig. 1:**
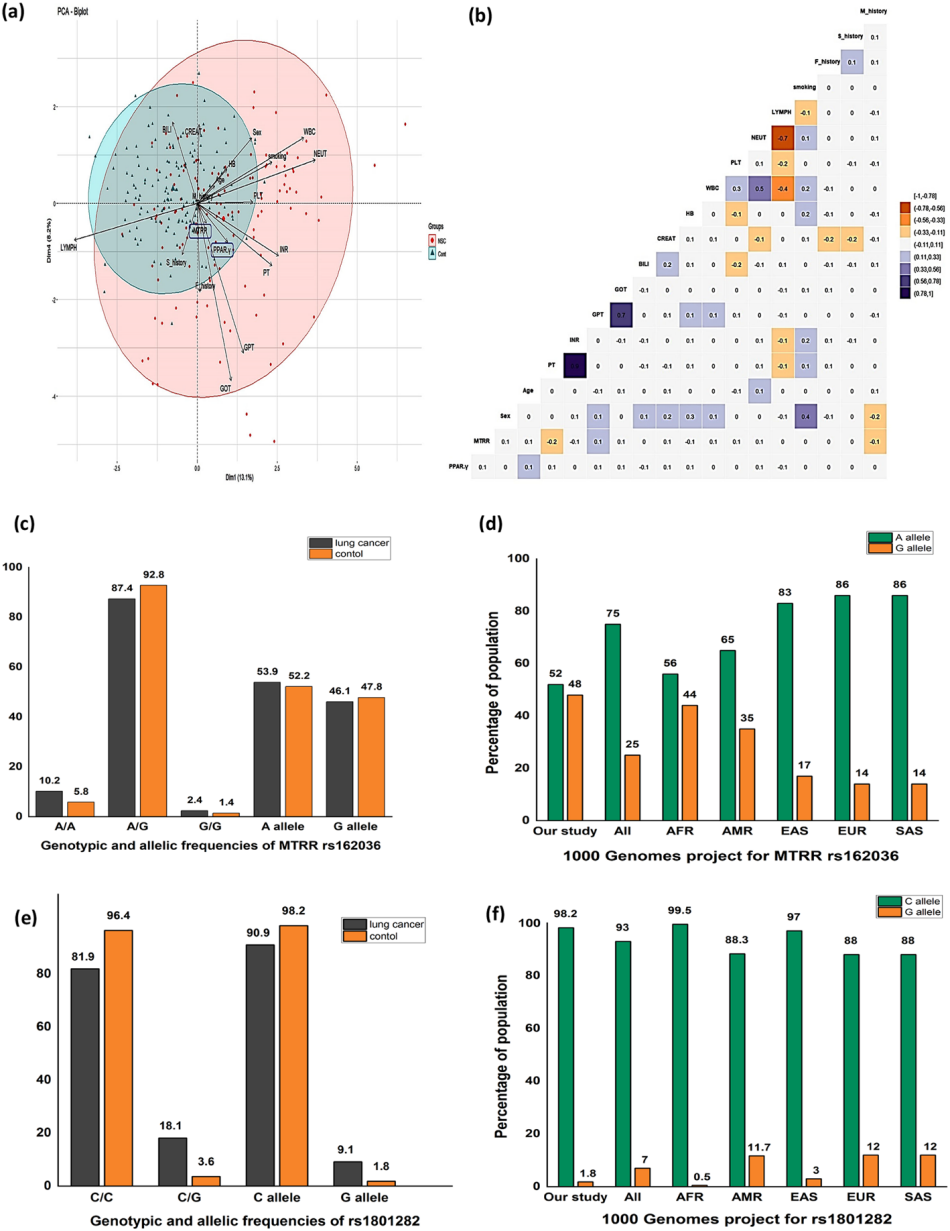
Allelic and genotypic frequencies of the study population. **a** Principal component analysis (PCA) of the study population showing distinct demarcation between NSCLC patients and cancer-free controls. Clinical characteristics and biochemical parameters were displayed by arrows, with elongated arrows showing more impact on separation. Visually, the *PPAR-γ* (rs1801282) variant was associated with increased risk of NSCLC. **b** Correlation matrix for the clinical characteristics and biochemical measurements of NSCLC patients. Pearson’s correlation test was applied with statistically significant associations were only displayed. **c** Genotype and allele frequencies of the *MTRR* (rs162036) variant among NSCLC patients and cancer-free controls. **d** Allelic frequencies of the *MTRR* (rs162036) variant in the current study compared to different populations based on the 1000 Genome project phase 3 (https://www.internationalgenome.org/). **e** Genotype and allele frequencies of the *PPAR-γ* (rs1801282) variant among NSCLC patients and cancer-free controls. **f** Allelic frequencies of the *PPAR-γ* (rs1801282) variant in the current study compared to different populations based on the 1000 Genome project phase 3. *AFR* Africa, *AMR* America, *EAS* East Asia, *EUR* Europe, *SAS* South Asia, *NSCLC* non-small cell lung cancer

Based on multivariate analysis, principal component analysis (PCA) was performed to explore overall patterns and relationships among clinical, biochemical, and genetic variables. As shown in (Fig. 1a), PCA clearly separated the study population into two distinct clusters corresponding to NSCLC patients and healthy controls, indicating that the combined variables contributed to effective group discrimination. Each arrow represents a clinical or biochemical parameter, with arrow length reflecting its relative contribution to the observed separation. Among these variables, the PPAR-γ (rs1801282) polymorphism showed a notable contribution to the separation of NSCLC cases from controls, suggesting an association with increased disease risk. (Fig. 1b**)** presents the correlation matrix among clinical characteristics and biochemical parameters within the NSCLC group. This analysis highlights the interrelationships between oxidative stress markers, hematological indices, and metabolic variables, providing an integrated view of how these parameters cluster together in NSCLC patients. Collectively supports the multivariate nature of NSCLC pathophysiology and demonstrates that genetic variation, in combination with biochemical and clinical factors, contributes to disease-associated patterns rather than acting as an isolated determinant.

### Association between MTRR (rs162036) polymorphism and NSCLC risk

Among NSCLC patients and controls, the 'AG' heterozygous genotype was the most frequent (87.4% and 92.8%, respectively), while the minor (G) allele occurred in 46.1% of cases and 47.8% of controls (Fig. 1c). Comparison with populations from the 1000 Genomes Project indicated similarity with African ancestry (Fig. 1d). The MTRR rs162036 polymorphism deviated from Hardy–Weinberg equilibrium in both groups; therefore, these results should be interpreted with caution.

The influence of the *MTRR* (rs162036) mutation on the incidence of NSCLC was analyzed under different genetic and allelic models. The distribution of the *MTRR* (rs162036) variant was not significantly different between NSCLC patients and controls in any of the models (*p* > 0.05). As summarized in Table 3, no significant association was observed between MTRR rs162036 and NSCLC risk under any genetic or allelic model (*p* > 0.05). Notably, this variant deviated from Hardy–Weinberg equilibrium in both cases and controls (*p* < 0.001), indicating that the null findings should be interpreted cautiously. This observation would require additional confirmation with a larger sample of controls** (**Table 3).

**Table 3:** Distribution of *MTRR* (rs162036) and *PPAR-γ* (rs1801282) polymorphisms among Egyptian patients with NSCLC compared to controls

Gene	Polymorphism genotype	Cases (n = 127) n (%)	Controls (n = 138) n (%)	Model	Genotype	OR (95% CI)	*P*^a^	Adjusted OR (95% CI)	*P*^b^
MTRR	AA	13 (10.2)	8 (5.8)			1.0		1.0	
	AG	111 (87.4)	128 (92.8)	Codominant	AG vs. AA	0.53 (0.21–1.34)	0.179	0.47 (0.17–1.28)	0.141
	GG ^†^	3 (2.4)	2 (1.4)		GG vs. AA	0.92 (0.13–6.78)	0.937	0.07 (0.002–3.25)	0.176
	GG + AG	114 (89.8)	130 (94.2)	Dominant	GG + AG vs. AA	0.54 (0.22–1.35)	0.187	0.47 (0.17–1.28)	0.139
	AA + AG	124 (97.6)	136 (98.6)	Recessive	GG vs. AA + AG	1.65 (0.27–10.0)	0.589	0.89 (0.11–7.47)	0.914
	AA + GG	16 (12.6)	10 (7.2)	Overdominant	AG vs. AA + GG	0.54 (0.24–1.24)	0.148	0.538 (0.22–1.35)	0.185
	A	137 (53.9)	144 (52.2)	Allelic	G vs. A	1.0		1.0	
	G	117 (46.1)	132 (47.8)			0.93 (0.66–1.31)	0.685	0.89 (0.61–1.3)	0.561
	HWE ^**c**^	X^2^ = 73.0, *P* < **0.001**	X^2^ = 101.6, *P* < **0.001**						
PPAR-γ	CC	104 (81.9)	133 (96.4)			1.0		1.0	
	CG	23 (18.1)	5 (3.6)			**5.88 (2.1–16.0)**	**0.001**	**6.74 (2.3–19.5)**	< **0.001**
	C	231 (90.9)	271 (98.2)						
	G	23 (9.1)	5 (1.8)			**5.39 (2.0–14.4)**	**0.001**	**6.1 (2.15–17.2)**	**0.001**
	HWE ^c^	X^2^ = 1.25, *P* = 0.53	X^2^ = 0.04, *P* = 0.97						

^a^*p* value for the genetic inheritance models obtained by logistic regression. b *p* value with adjusted age, sex, smoking, and family history. c Hardy–Weinberg equilibrium (HWE) was assessed using the Chi-square test. †The MTRR rs162036 polymorphism deviated from HWE in both cases and controls (*p* < 0.001), which may reflect factors such as selection bias, population structure, or technical limitations related to genotyping. Therefore, associations involving this variant should be interpreted with caution. In contrast, PPAR-γ rs1801282 conformed to HWE in both groups (*p* > 0.05); NA not applicable; Bold values express the *p* < 0.05. Estimates for rare genotypes, particularly the GG genotype of MTRR rs162036, should be interpreted with caution due to the very small number of observations, which may result in unstable odds ratio estimates

### Association between PPAR-γ (rs1801282) polymorphism and NSCLC risk

*PPAR-γ* (rs1801282) frequencies were coordinated with Hardy–Weinberg equilibrium (*p* > 0.05) in both cases and controls. *PPAR-γ* minor allele frequency (G allele) was about 9.1% with NSCLC patients and 1.8% with cancer-free controls. The rs1801282 genotypes revealed a lack of the uncommon 'GG' genotype in both cases and controls (Fig. 1e). Furthermore, *PPAR-γ* frequencies of both the (C) and (G) alleles in this study were parallel to those observed in African and East Asian populations in the 1000 Genome Project (Fig. 1f).

As demonstrated in the dominant model, individuals having the heterozygous genotype 'CG' were more susceptible to NSCLC than those with the normal-type genotype 'CC' carriers (adjusted, OR = 6.74, *p* < 0.001). In addition, the mutant allele 'G' of rs1801282 raised the likelihood of NSCLC by 6.1-fold in the allele model (adjusted, OR = 6.1, *p* = 0.001) **(**Table 3).

### Stratification analysis of MTRR and PPAR-γ variants and NSCLC risk

The stratification of *MTRR* (rs162036) was executed by age, gender, smoking, and histological subtypes of NSCLC. The results indicated that rs162036 exhibited a protective role against NSCLC incidence across genotypic models; however, no statistically significant result was reached (Table 4).

**Table 4:** Association between *MTRR* (rs162036) polymorphism and lung cancer under different stratification analyses

Variable	Cases/ Controls			Adjusted OR (95% CI); *P* ^a^				
	AA	AG	GG	AG vs. AA	GG vs. AA	GG + AG vs. AA	GG vs. AA + AG	AG vs. AA + GG
*Sex*								
Female	6/3	45/42	0/1	0.54 (0.12–2.4); 0.431	NA (0.0–NA); 0.999	0.61 (0.13–2.8); 0.523	NA (0.0–NA); 1.0	0.87 (0.21–3.6); 0.854
Male	7/5	66/86	3/1	0.43 (0.11–1.6); 0.214	0.89 (0.03–26.7); 0.947	0.42 (0.11–1.6); 0.202	2.15 (0.14–32.3); 0.581	0.42 (0.12–1.42); 0.16
*Age*								
< 55	6/6	51/67	1/1	0.75 (0.2–2.6); 0.654	NA (0.0–NA); 0.999	0.74 (0.21–2.65); 0.64	NA (0.0–NA); 0.999	0.84 (0.24–2.91); 0.782
> = 55	7/2	60/61	2/1	0.24 (0.04–1.4); 0.116	NA (0.0–NA); 0.999	0.24 (0.04–1.42); 0.116	1.19 (0.09–14.4); 0.889	0.33 (0.07–1.45); 0.144
*Smoking*								
Nonsmoker	7/7	54/96	1/2	0.59 (0.18–1.96); 0.392	NA (0.0–NA); 0.999	0.58 (0.18–1.92); 0.374	NA (0.0–NA); 0.999	0.78 (0.25–2.45); 0.676
Smoker	6/1	57/32	2/0	0.27 (0.03–2.4); 0.242	NA (0.0–NA); 0.999	0.27 (0.03–2.42); 0.241	NA (0.0–NA); 0.999	0.22 (0.02–1.88); 0.165
*Histological types*								
Adenocarcinoma	10/8	87/129	2/2	0.43 (0.15–1.2); 0.115	0.26 (0.01–5.9); 0.403	0.43 (0.15–1.24); 0.117	1.15 (0.14–9.6); 0.90	0.48 (0.19–1.26); 0.137
Squamous cell carcinoma	1/8	12/128	0/2	0.25 (0.02–2.7); 0.262	NA (0.0–NA); 0.999	0.23 (0.02–2.56); 0.233	NA (0.0–NA); 0.999	0.29 (0.02–3.2); 0.317
Large cell carcinoma	2/8	12/128	1/2	0.65 (0.06–6.9); 0.725	NA (0.0–NA); 0.999	0.55 (0.05–6.1); 0.626	NA (0.0–NA); 0.999	0.63 (0.06–6.6); 0.697

^a^ *p* value for the genetic models obtained by logistic regression with adjusted age, sex, smoking, and family history

*OR* odds ratio, *CI* confidence interval 95%; *NA* not applicable;* p* > 0.05: non-significant

Astonishingly, *PPAR-γ* (rs1801282) revealed that males bearing the 'CG' heterozygote genotype were more susceptible to NSCLC (adjusted, CG vs. CC, OR = 4.03, 95% CI: 1.22–13.35; *p* = 0.023). Elderly patients over 55 years of age with the *PPAR*-*γ* 'CG' genotype had a higher contingency of NSCLC risk (adjusted, CG vs. CC, OR = 18.75, 95% CI: 2.2–158.1; *p* = 0.007). The *PPAR*-*γ* 'CG' genotype also had a significant effect on increasing NSCLC incidence in nonsmokers (for CG vs. CC, adjusted, OR = 7.01, *p* = **0.00**2). When segregating by histological subtypes of NSCLC, it was shown that the *PPAR-γ* heterozygote remarkably increased the risk of AC (adjusted, CG vs. CC, OR = 7.46, 95% CI: 2.5–22.45; *p* < 0.001) and SCC (adjusted, CG vs. CC, OR = 14.3, 95% CI: 1.6–128.9; *p* = 0.018) **(**Table 5). These subgroup-specific associations, particularly those with large effect sizes, should be considered exploratory and hypothesis-generating rather than precise estimates of genetic risk.

**Table 5:** Association between *PPAR-γ* (rs1801282) polymorphism and lung cancer under different stratification analyses

Variable	Cases/ Controls		Adjusted OR (95% CI); *P* ^a^
	CC	CG	CG vs. CC
*Sex*			
Female	41/46	10/0	NA (0.0–NA); 0.999
Male	63/87	13/5	**4.03 (1.22**–**13.35); 0.023**
*Age*			
< 55	50/70	8/4	3.67 (0.98–13.67); 0.053
> = 55	54/63	15/1	**18.75 (2.2**–**158.1); 0.007**
*Smoking*			
Nonsmoker	50/101	12/4	**7.01 (2.0–24.5); 0.002**
Smoker	54/32	11/1	5.98 (0.72–49.8); 0.098
*Histological types*			
Adenocarcinoma	79/134	20/5	**7.46 (2.5**–**22.45); < 0.001**
Squamous cell carcinoma	11/133	2/5	**14.3 (1.6–128.9); 0.018**
Large cell carcinoma	14/133	1/5	2.36 (0.22–25.5); 0.477

^a^*p* value obtained by logistic regression with adjusted age, sex, smoking, and family history

*OR* odds ratio, *CI* confidence interval 95%; *NA* not applicable; Bold values express the *p* < 0.05

### Association of MTRR and PPAR-γ variants and clinical characteristics of NSCLC patients

The relations between the dominant and allele models of *MTRR* (rs1801282) and the laboratory, clinical, and pathological aspects of NSCLC were statistically non-significant (*p* > 0.05) (Table S1). In addition, the genotype and allele models of *PPAR-γ* (rs1801282) showed no significant connection with the clinicopathological data of NSCLC (*p* > 0.05) (Table S2).

### In silico* data analysis*

**Fig. 2:**
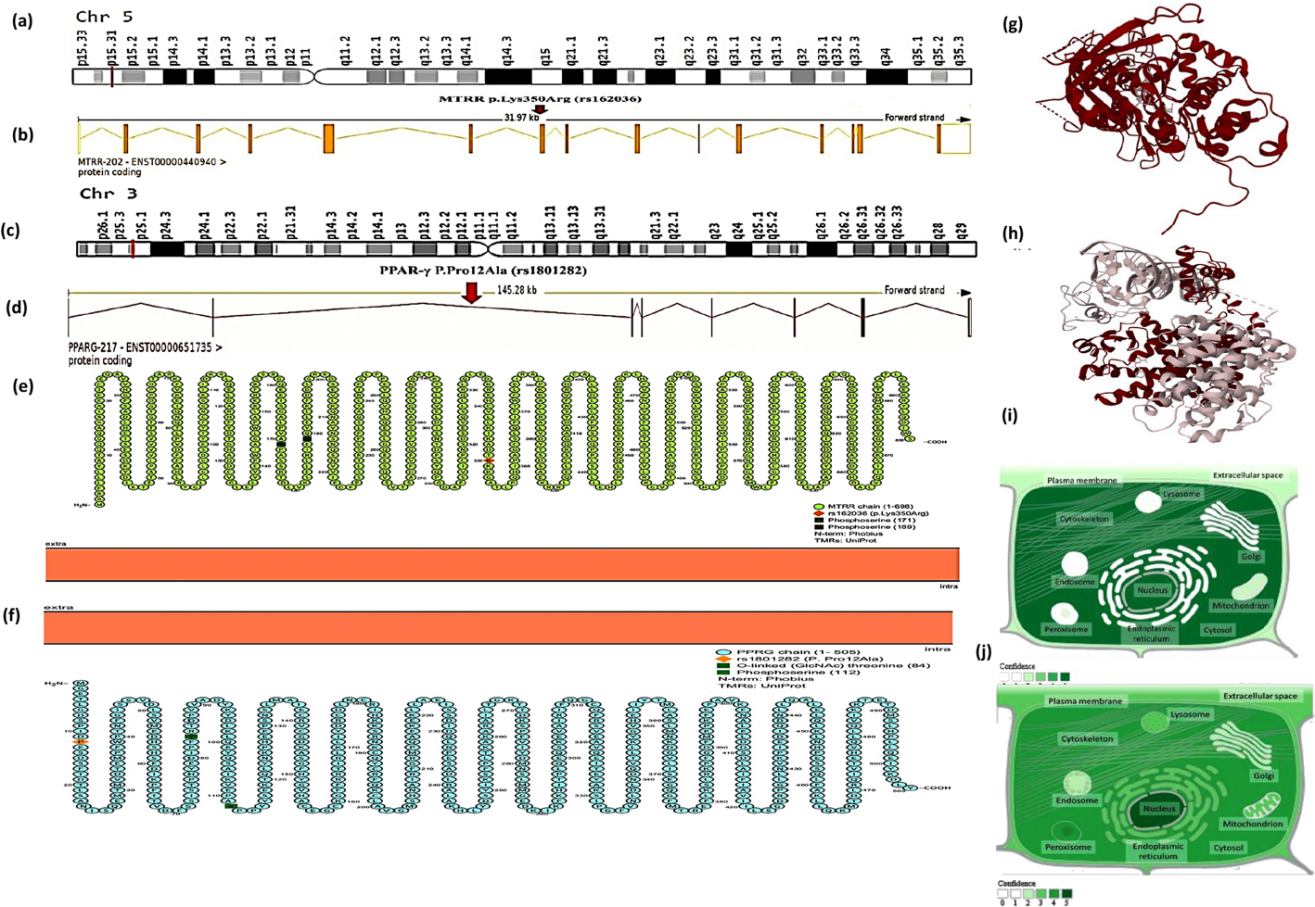
The computational bioinformatics frameworks of the *MTRR* and *PPAR-γ* genes.** a** The *MTRR* gene is located at chromosome 5p15.31 and spans 50,255 bases (chr5: 7,850,859–7,901,113) along the plus strand. **b** The genomic structure of the main transcript (MTRR -202) contains 15 exons with 14 introns. The missense *MTRR* *p*.Lys350Arg (rs162036) variant is located at chromosome 5:7,885,846 within the seventh exon of the MTRR -202 transcript. **c** The *PPAR-γ* gene is located at chromosome 3p25.2 and spans 146,977 bases (chr3: 12,287,368–12,434,344) along the plus strand. **d** The genomic structure of the main transcript (PPAR-γ -217) contains 8 exons with 7 introns. The *PPAR-γ* P.Pro12Ala (rs1801282) missense variant is located on the chromosome 3:12,351,626 within the second intron of the PPAR-γ -217 transcript (https://www.ensembl.org/). **e** Amino acid residues of the MTRR protein and its domains displaying the position of *MTRR* *p*.Lys350Arg variant. **f** Amino acid residues of the PPAR-γ protein and its domains showing the site of *PPAR-γ* P.Pro12Ala variant (https://wlab.ethz.ch/protter/). **g** 3D structure of the MTRR protein. **h** 3D structure of the PPAR-γ protein (https://www.rcsb.org/). **i** Subcellular localization of the MTRR protein, with darker colors showing more copiousness. **j** Subcellular localization of the PPAR-γ protein, with darker colors indicating more abundance (https://compartments.jensenlab.org/). (Data source: Ensembl.org, NCBI database, Protter database, Protein Data Bank database, Compartment database)

**Fig. 3:**
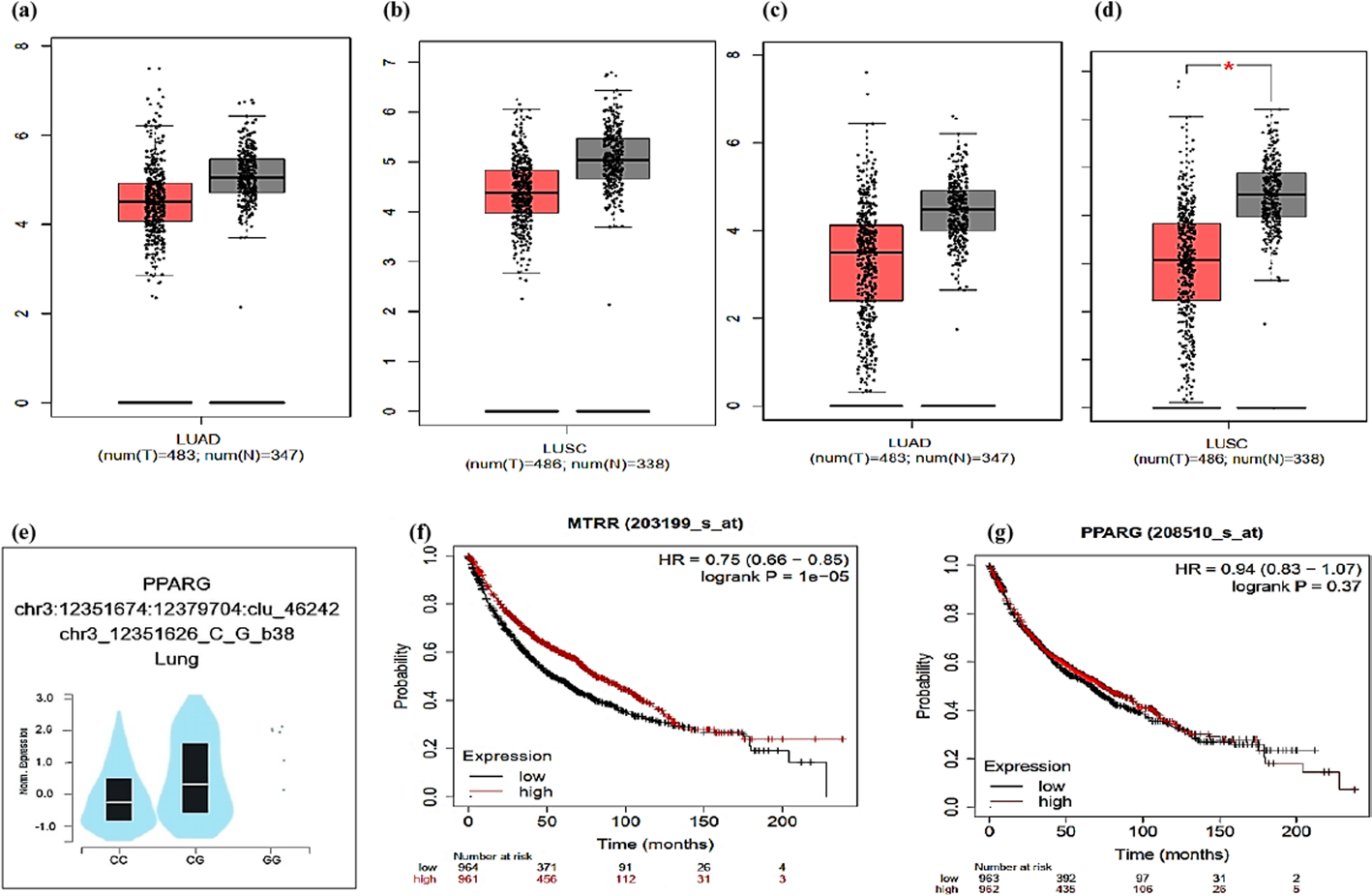
Prediction of the expression and prognosis of *MTRR* and *PPAR-γ* gene in lung cancer. **a** Expression of *MTRR* gene in LUAD (*n* = 483) and para-tumor tissues (*n* = 347). **(b)** Expression of *MTRR* gene in LUSC (*n* = 486) and para-tumor tissues (*n* = 338).** c** Expression of *PPAR-γ* gene in LUAD (*n* = 483) and para-tumor tissues (*n* = 347). **d** Expression of *PPAR-γ* gene in LUSC (*n* = 486) and para-tumor tissues (*n* = 338). The *Y*-axis is the log-scale of log 2(TPM + 1) (*TPM* Transcripts Per Million). The box plots show the interquartile range (IQR), median (bar in box), * *p* < 0.01 designates statistical significance (http://gepia.cancer-pku.cn/). **f** Expression of *PPAR-γ* P.Pro12Ala (rs1801282) in normal lung tissues (*p* = 4.2 × 10^–13^) using GTEx database (https://www.gtexportal.org/). **f** Survival analysis in high and low *MTRR* expression. **g** Survival analysis in high and low *PPAR-γ* expression. The *Y*-axis is survival rate. Red line represents high expression, and black line represents low expression; *P* value < 0.05 was considered statistically significant; *HR* Hazard ratio (https://kmplot.com/analysis/). Data source: GEPIA database, GTEx database, Kaplan‐Meier plotter database. (Lung squamous cell carcinoma [LUSC]; lung adenocarcinoma [LUAD])

All bioinformatics data of the *MTRR* and *PPAR-γ* genes are depicted in Figs. 2, 3. The *MTRR* gene [ENSG00000124275], also referred to as MSR or cblE, is located on the forward strand of chromosome 5p15.31 (chr5: 7,850,859–7,901,113) and covers around 50,255 bases. There are 25 splice variants in this gene, with the principal transcript, MTRR-202 (ENST00000440940.7), containing 15 exons and 14 introns. The *MTRR* Lys350Arg (rs162036) missense variation is found in chromosome 5:7,885,846 with a minor allele frequency (G) of 0.25 [Sources: Ensembl.org (https://www.ensembl.org/); Human Genome Assembly (GRCh38), NCBI (https://www.ncbi.nlm.nih.gov/)]. The *MTRR* gene codes for methionine synthase reductase (EC 2.1.1.135). In animals, methionine is an essential amino acid, required for protein synthesis and functionally important in 1-carbon metabolism. The MTRR protein (Q9UBK8) involves 698 AAs and has a molecular weight of 77,674 Da [Sources: Uniprot database (https://www.uniprot.org/); Protter database (https://wlab.ethz.ch/protter/)]. The MTRR enzyme is predominantly located in the nucleus, cytosol, and cytoskeleton [Source: Database of Cellular Compartments (https://compartments.jensenlab.org/)] (Fig. 2).

The *PPAR-γ* gene [ENSG00000132170] is also known by several aliases such as CIMT1, NR1C3, GLM1, PPARG1, PPARG2, PPARG5, and PPARgamma. This gene is extended on the chromosome 3 arm (3p25.2), covering 146,977 bases along the forward strand (chr3: 12,287,368–12,434,344), and comprises 34 splice variants. The primary transcript, PPAR-γ-217 (ENST00000651735.1), has 8 exons and 7 introns. The *PPAR-γ* Pro12Ala (rs1801282) missense variation is found in chromosome 3:12,351,626 and has a minor allele frequency (G) of 0.07 [Sources: Ensembl.org (https://www.ensembl.org/); Human Genome Assembly (GRCh38), NCBI (https://www.ncbi.nlm.nih.gov/)]. The *PPAR-γ* gene assembles the peroxisome proliferator-activated receptor gamma, which triggers peroxisome proliferation and adipocyte differentiation. The PPAR-γ protein (P37231) encompasses 505 AAs and a molecular weight of 57,620 Da [Sources: Uniprot database (https://www.uniprot.org/); Protter database (https://wlab.ethz.ch/protter/)]. The nuclear receptor PPAR-γ is mostly found in the nucleus, cytosol, and peroxisomes [Source: Database of Cellular Compartments (https://compartments.jensenlab.org/)] (Fig. 2).

## Discussion

The wide genetic variation in NSCLC, particularly single nucleotide variants, has garnered significant interest in recent years as crucial etiological factors. Thus far, few studies have explored the effects of *PPAR-γ* rs1801282 and *MTRR* rs162036 on NSCLC risk among different ethnicities. This case–control study is the first to clarify the association between *PPAR-γ* and *MTRR* genetic variants and NSCLC susceptibility in the Egyptian population.

Intrinsically, our work identified that *PPAR-γ* rs1801282 significantly elevated the risk of developing NSCLC in the genotypic (*p* < 0.001) and allelic models (*p* = 0.001). The effect of the *PPAR-γ* (CG) genotype was modified by gender, age, smoking habits, and histological subtypes of NSCLC. The expression of the *PPAR-γ* gene is significantly higher in lung squamous cell carcinoma (LUSC) (*p* < 0.01), whereas *PPAR-γ* Pro12Ala is notably expressed in normal lung tissues (*p* = 4.2 × 10^–13^). Lung cancer patients also revealed improved survival rate with increased *MTRR* expression (*P* = 1 × 10^–5^) [Sources: GEPIA (http://gepia.cancer-pku.cn/), GTEx (https://www.gtexportal.org/), and Kaplan–Meier Plotter databases (https://kmplot.com/analysis/)] (Fig. 3).

**Fig. 4:**
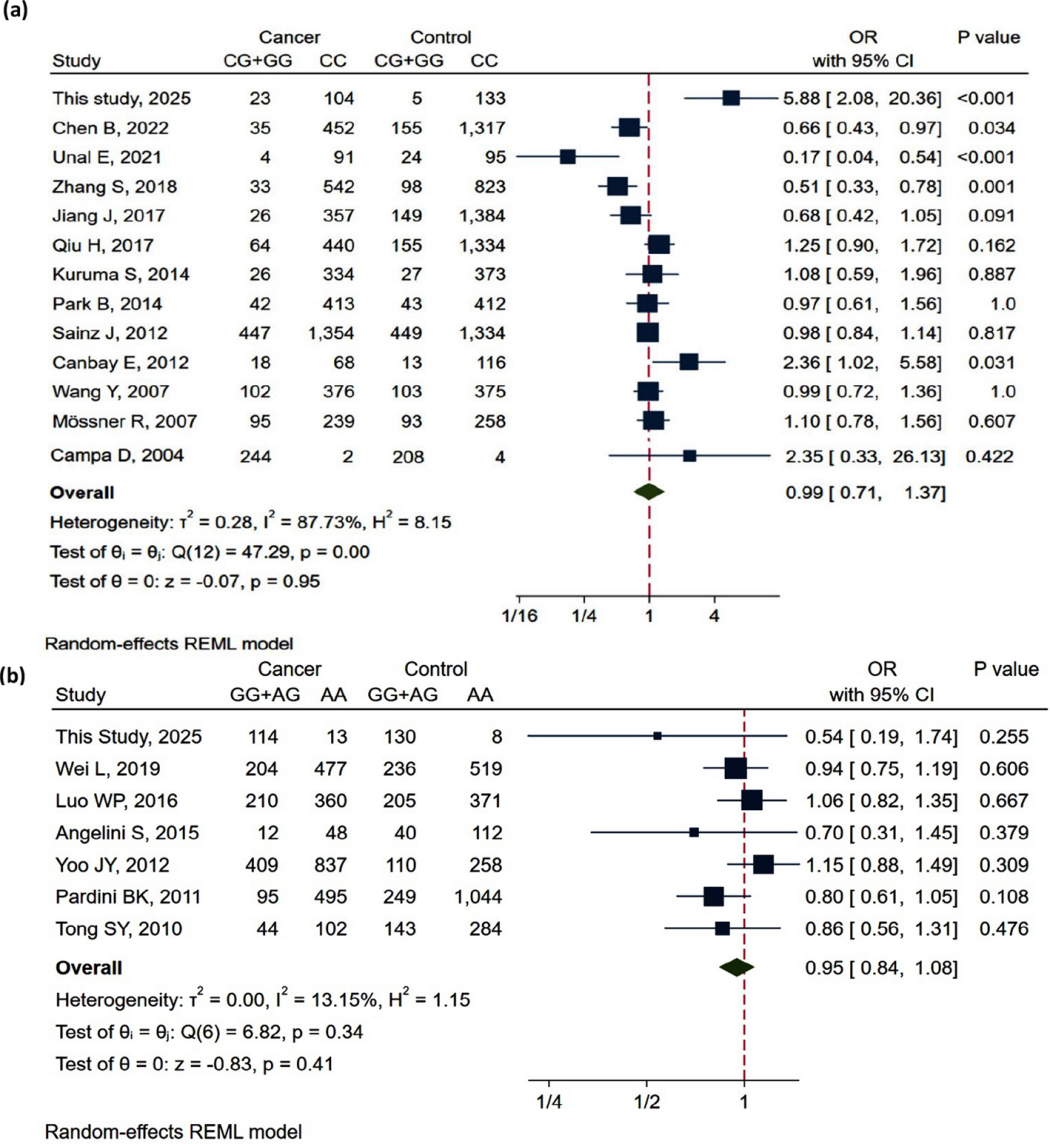
Forest plots of the pooled data for the association between *PPAR-γ* (rs1801282) and *MTRR* (rs162036) variants and different types of cancers. **a** *PPAR-γ* (rs1801282) under the dominant model (CG + GG vs. CC). **b** *MTRR* (rs162036) under the dominant model (GG + AG vs. AA)

Our team encountered only one report investigating the connotation of the *PPAR-γ* (rs1801282) variant with the occurrence of lung cancer [36]. To expand upon this, we conducted extensive research on previous studies published between 2004 to 2025 to perform a pooled analysis assessing the relationship between the *PPAR-γ* (rs1801282) variant and various cancer types among different ethnic groups (Table S3). In accordance with our findings, four reports have confirmed the effect of the *PPAR-γ* (rs1801282) variant on the susceptibility for hepatocellular carcinoma [37], gastric cancer [38, 39], and breast cancer [28]. However, several studies documented a non-significant association of *PPAR-γ* (rs1801282) variant with the incidence of multiple tumors, involving NSCLC in Norwegian cases [36], melanoma in German cases [40], colorectal cancer in both German [41] and Eastern Chinese cases [42], breast cancer in American [43] and Korean cases [44], pancreatic cancer in Japanese cases [45], and esophageal squamous-cell cancer in Eastern Chinese cases [46]. Overall, the findings identified no significant difference between the *PPAR-γ* (rs1801282) variant and cancer, as exemplified in the forest plot **(**Fig. 4**)**. The large heterogeneity the collected reports might be attributed to disparities in race, cancer types, and environmental factors.

In addition, this study revealed that the *PPAR-γ* (CG) genotype showed a significant association with NSCLC incidence in males, people older than fifty-five years old, nonsmokers, and particularly in the AC and SCC subtypes. In agreement with our results, Chen et al. [39] conveyed that the *PPAR-γ* rs1801282 SNP was correlated with a higher susceptibility to gastric cancer in the Eastern Chinese population, particularly with subgroups aged ≥ 61 and nonsmokers. Besides, a Turkish case–control study demonstrated that the *PPAR-γ* Pro12Pro genotype was associated with an increased risk of breast cancer compared with Ala12 allele carriers (*p* < 0.001). The multivariate regression analysis in their study confirmed that the Pro12Pro genotype (*p* < 0.011), T161 allele (*p* < 0.001), smoking (*p* = 0.019), and advanced a (> 60 years) (*p* = 0.007) were risk factors for breast cancer [28]. Although an a priori power calculation indicated adequate overall statistical power for the primary analyses, an important limitation of the present study is the small number of individuals within certain genotype categories, particularly those involving rare alleles. Low minor allele frequencies inevitably reduce the effective sample size in stratified and subgroup analyses, leading to unstable effect estimates, inflated odds ratios, wide confidence intervals, and, in some models, non-estimable values. This phenomenon is evident in some stratified results, such as the markedly elevated odds ratio observed for the PPAR-γ rs1801282 variant in individuals older than 55 years. Such exaggerated effect sizes are a recognized statistical consequence of sparse data rather than definitive evidence of strong biological effects. Therefore, these stratified findings should be interpreted as exploratory and hypothesis-generating, and not as precise estimates of genetic risk. Larger studies with higher representation of rare genotypes or pooled multi-center analyses are required to obtain more stable and reliable risk estimates.

PPAR-γ is a ligand-activated nuclear receptor that heterodimerizes with RXRα and regulates transcription of genes involved in metabolism, inflammation, and cell proliferation [47, 48]. PPAR-γ is expressed in various malignancies involving colon, prostate, breast, lung, and prostate cancers, where it has a tumor-suppressive function in most cancers [23, 49]. In cancer models, PPAR-γ activation has been linked to antiproliferative and pro-apoptotic effects through modulation of apoptosis-related genes, cell cycle regulators, angiogenesis, and PI3K/Akt signaling [50–56]. In NSCLC, experimental evidence suggests that PPAR-γ signaling may influence tumor growth and therapeutic response; however, the functional impact of the Pro12Ala (rs1801282) variant remains incompletely understood and likely context-dependent [25, 55].

On the other hand, the *MTRR* rs162036 variant was non significantly different between NSCLC patients and controls in the Egyptian cohorts. Nevertheless, no previous association studies have assessed the effect of this variant on any disease within the Egyptian population. Besides no prior studies have examined the relationship between this variant and NSCLC in other populations. Therefore, we report the distribution of MTRR rs162036 and PPAR-γ rs1801282 genotypes among different cancer types based on our study cohort and reference datasets, providing comparative insights without conducting a formal meta-analysis (Table S4). A number of case-controlled studies have demonstrated a non-significant association between the *MTRR* rs162036 SNP and the risk of colorectal [14], gastric [57–59], prostate [59], and cervical [60] cancerous diseases. Contrarily, two studies conducted in Chinese populations have suggested that the *MTRR* rs162036 variant may influence susceptibility to breast cancer [20] and acute lymphoblastic leukemia [18]. The results are summarized in a forest plot, which eventually declares no overall association between *MTRR* rs162036 and the development of cancer (Fig. 4). The relatively high odds ratios observed in our study, particularly in stratified analyses, may reflect population-specific genetic architecture, allele frequency differences, environmental exposures, and statistical instability from small subgroup sizes rather than stronger biological effects.

The deviation from Hardy–Weinberg equilibrium (HWE) observed for the MTRR rs162036 polymorphism in both NSCLC cases and controls. Deviation from HWE in the control group may reflect several factors, including selection bias, subtle population These subgroup-specific associations, particularly those with large effect sizes, should be considered exploratory and hypothesis-generating rather than precise estimates of genetic risk These subgroup-specific associations, particularly those with large effect sizes, should be considered exploratory and hypothesis-generating rather than precise estimates of genetic risk, or locus-specific genotype distribution characteristics. In the current cohort, rs162036 exhibited an unusually high frequency of the heterozygous AG genotype and a very low frequency of the GG genotype, a pattern that can inflate chi-square values and lead to apparent HWE deviation, particularly in studies with moderate sample sizes. To minimize the possibility of genotyping errors, all samples were analyzed using a validated T-ARMS-PCR protocol, and approximately 10% of samples were randomly re-genotyped with complete concordance. Although true biological or evolutionary selection cannot be entirely excluded, the lack of any significant association between rs162036 and NSCLC risk across all genetic models suggests that the observed HWE deviation did not produce a false-positive result. Nevertheless, associations involving this variant should be interpreted cautiously and warrant validation in larger, independent populations. Alternatively, the observed deviation may reflect sampling imbalance or population structure. Given the absence of any significant association between rs162036 and NSCLC risk across all genetic models, these findings should be considered non-conclusive and interpreted cautiously.

Based on the aforementioned outcomes, this study suggests that the *PPAR-γ* Pro12Ala variant is associated with the development of NSCLC. However, certain limitations of the study should be taken into account, such as its single-center design and the lack of data on protein levels. Therefore, further research on *PPAR-γ* variants across larger populations and ethnically diverse backgrounds is strongly recommended in order to reinforce our findings and provide other insights into this area.

In the context of recent GWAS and meta-analyses on NSCLC genetics, our findings support the complementary role of candidate-gene approaches in elucidating population-specific risk variants. While GWAS provide robust identification of common low-effect loci, they may overlook functional variants with moderate effects, particularly in genes involved in metabolic regulation and redox balance, such as PPAR-γ. Moreover, genetic associations can vary substantially across ethnicities due to differences in allele frequencies, linkage disequilibrium patterns, and environmental exposures, including smoking behavior. Therefore, our observation of a significant association between PPAR-γ rs1801282 and NSCLC risk in Egyptians may reflect ethnicity-specific genetic susceptibility that is not captured in large GWAS datasets. Our elevated OR contrasts modest effects in non-lung metas, potentially due to Egyptian-specific modifiers These findings highlight the importance of integrating candidate-gene studies with GWAS evidence to achieve a more comprehensive understanding of NSCLC genetic risk, particularly in populations that remain underrepresented in global genomic research [61].

Smoking exposure was recorded as a binary variable (smoker vs. non-smoker), and detailed information on pack-years, duration, type of tobacco use, passive smoking, occupational exposures, and comorbid conditions was not available. Although smoking status was included as a covariate in multivariate and stratified analyses, residual confounding by unmeasured environmental and lifestyle factors cannot be excluded. Therefore, the observed genetic associations should be interpreted with caution, as they may be partially influenced by factors not captured in the current dataset. The large number of statistical comparisons across genetic models and subgroups, without formal correction for multiple testing. This may increase type I error, making some marginally significant associations potentially false positives. Given the exploratory design, results should be interpreted cautiously and considered hypothesis-generating until confirmed in independent cohorts. Future studies should incorporate haplotype-based and multi-locus analyses of PPAR-γ, MTRR, and related pathway genes, as haplotypes may better capture combined genetic effects and gene–gene interactions than single-SNP analyses, Finally, given the modest sample size and single-center design, further multicenter studies with larger, well-characterized cohorts are warranted to corroborate and extend these findings. Future studies integrating environmental exposure data and patient-specific transcriptomic profiling will be essential to clarify potential gene environment interactions involving PPAR-γ polymorphisms.

## Conclusion

In conclusion, this exploratory case–control study provides preliminary evidence suggesting a potential association between the CG heterozygous genotype and the G allele of the PPAR-γ rs1801282 polymorphism and NSCLC susceptibility in an Egyptian population. The observed associations remained significant after adjustment for major covariates, including age, sex, smoking status, and family history; however, residual confounding related to unmeasured environmental and lifestyle factors cannot be excluded, and the statistical stability of several subgroup analyses was limited by small genotype counts. Therefore, these findings should be interpreted cautiously and considered hypothesis-generating rather than definitive. In contrast, no significant association was observed between the MTRR rs162036 polymorphism and NSCLC risk. Given the methodological limitations of the study, including sample size constraints, limited environmental exposure characterization, and Hardy–Weinberg disequilibrium for MTRR, further large-scale, multi-center studies incorporating detailed exposure assessment and independent genotyping approaches are required to validate these results.

## Supplementary Information

Supplementary Material 1.

## Data Availability

The PPAR-γ variant dataset used in this study is available from the ClinVAR database (https://www.ncbi.nlm.nih.gov/clinvar/variation/130019/?oq=((135465[AlleleID]))&amp;m=NM_138711.6(PPARG):c.-8-28078C%3EG). The PPAR-γ variant dataset used in this study is available from the ClinVAR database (https://www.ncbi.nlm.nih.gov/clinvar/variation/130019/?oq=((135465[AlleleID]))&m=NM_138711.6(PPARG):c.-8-28078C%3EG).

## References

[1] Siegel RL, Miller KD, Wagle NS, Jemal A. Cancer statistics, 2023. CA Cancer J Clin. 2023;73:17–48. 36633525 10.3322/caac.21763

[2] Fu Y, Liu J, Chen Y, Liu Z, Xia H, Xu H. Gender disparities in lung cancer incidence in the United States during 2001–2019. Sci Rep. 2023;13:12581. 37537259 10.1038/s41598-023-39440-8 PMC10400573

[3] Sung H, Ferlay J, Siegel RL, Laversanne M, Soerjomataram I, Jemal A, et al. Global cancer statistics 2020: GLOBOCAN estimates of incidence and mortality worldwide for 36 cancers in 185 countries. CA Cancer J Clin. 2021;71:209–49. 33538338 10.3322/caac.21660

[4] Sayed RE, Abdul-Sater Z, Mukherji D. Cancer care during war and conflict. In: Al-Shamsi HO, Abu-Gheida IH, Iqbal F, Al-Awadhi A, editors. Cancer in the Arab world. Singapore: Springer; 2022.

[5] Salim EI, Jazieh AR, Moore MA. Lung cancer incidence in the Arab league countries: risk factors and control. Asian Pac J Cancer Prev. 2011;12(1):17–34. 21517227

[6] Gouvinhas C, De Mello RA, Oliveira D, Castro-Lopes JM, Castelo-Branco P, Dos Santos RS, et al. Lung cancer: a brief review of epidemiology and screening. Future Oncol. 2018;14:567–75. 29417838 10.2217/fon-2017-0486

[7] Duma N, Santana-Davila R, Molina JR. Non–small cell lung cancer: epidemiology, screening, diagnosis, and treatment. Mayo Clin Proc. 2019;94:1623–40. 31378236 10.1016/j.mayocp.2019.01.013

[8] Lemjabbar-alaoui H, Hassan OUI, Yang Y, Buchanan P. Lung cancer : biology and treatment options. Biochimica et Biophysica Acta (BBA). 2015;1856:189–210. 26297204 10.1016/j.bbcan.2015.08.002 PMC4663145

[9] Nasim F, Sabath BF, Eapen GA. Lung cancer. Med Clin North Am. 2019;103:463–73. 30955514 10.1016/j.mcna.2018.12.006

[10] Zienolddiny S, Skaug V. Single nucleotide polymorphisms as susceptibility, prognostic, and therapeutic markers of nonsmall cell lung cancer. Lung Cancer. 2015;2011(3):1–14. 10.2147/LCTT.S13256 PMC5312489 28210120

[11] Lyon P, Strippoli V, Fang B, Cimmino L. B vitamins and one-carbon metabolism: implications in human health and disease. Nutrients. 2020;12:1–24. 10.3390/nu12092867 PMC7551072 32961717

[12] Menezo Y, Elder K, Clement A, Clement P. Folic acid, folinic acid, 5 methyl tetrahydrofolate supplementation for mutations that affect epigenesis through the folate and one-carbon cycles. Biomolecules. 2022;12:197. 35204698 10.3390/biom12020197 PMC8961567

[13] Sadhukhan S, Paul S, Bankura B, Munian D, Ghosh S, Das M. Genetic analysis of MTR and MTRR gene polymorphisms in healthy mothers from eastern part of India. Int J Res Dev Pharm L Sci. 2017;7:2881–5.

[14] Pardini B, Kumar R, Naccarati A, Prasad RB, Forsti A, Polakova V, et al. MTHFR and MTRR genotype and haplotype analysis and colorectal cancer susceptibility in a case – control study from the Czech Republic. Mutat Res. 2011;721:74–80. 21211571 10.1016/j.mrgentox.2010.12.008

[15] Yadav U, Kumar P, Rai V. Distribution of methionine synthase reductase (MTRR) gene A66G polymorphism in Indian population. Indian J Clin Biochem. 2021;36:23–32. 33505124 10.1007/s12291-019-00862-9 PMC7817751

[16] Zhong G, Luo X, Li J, Liao Y, Gui G, Sheng J. MTRR rs1532268 polymorphism and gastric cancer risk: evidence from a meta-analysis. J Int Med Res. 2022;50:3000605221097486. 35579185 10.1177/03000605221097486 PMC9127855

[17] Lu YT, Gunathilake M, Lee J, Choi IJ, Kim YI, Kim J. Riboflavin intake, MTRR genetic polymorphism (rs1532268) and gastric cancer risk in a Korean population: a case-control study. Br J Nutr. 2022;127:1026–33. 34078503 10.1017/S0007114521001811

[18] Li M, Kong XY, Wang SM. Analysis of the frequency distribution of five single-nucleotide polymorphisms of the MTRRgene in a Chinese pediatric population with acute lymphoblastic leukemia. Pharmacotherapy. 2022;42:442–52. 35434830 10.1002/phar.2685

[19] Gautam KA, Raghav A, Sankhwar SN, Singh R, Tripathi P. Genetic polymorphisms of gene methionine synthase reductase (MTRR) and risk of urinary bladder cancer. Asian Pac J Cancer Prev. 2023;24:1137–41. 37116134 10.31557/APJCP.2023.24.4.1137 PMC10352732

[20] Luo WP, Li B, Lin FY, Yan B, Du YF, Mo XF, et al. Joint effects of folate intake and one-carbon-metabolizing genetic polymorphisms on breast cancer risk: a case-control study in China. Sci Rep. 2016;6:29555. 27404801 10.1038/srep29555 PMC4941723

[21] Harmon GS, Lam MT, Glass CK. PPARs and lipid ligands in inflammation and metabolism. Chem Rev. 2011;111:6321–40. 21988241 10.1021/cr2001355 PMC3437919

[22] Hassan FU, Nadeem A, Li Z, Javed M, Liu Q, Azhar J, et al. Role of peroxisome proliferator‐activated receptors (PPARs) in energy homeostasis of dairy animals: exploiting their modulation through nutrigenomic interventions. Int J Mol Sci. 2021;22:12463. 34830341 10.3390/ijms222212463 PMC8619600

[23] Sun J, Yu L, Qu X, Huang T. The role of peroxisome proliferator-activated receptors in the tumor microenvironment, tumor cell metabolism, and anticancer therapy. Front Pharmacol. 2023;14:1184794. 37251321 10.3389/fphar.2023.1184794 PMC10213337

[24] Carvalho MV, Gonçalves-De-albuquerque CF, Silva AR. PPAR gamma: from definition to molecular targets and therapy of lung diseases. Int J Mol Sci. 2021;22:805. 33467433 10.3390/ijms22020805 PMC7830538

[25] Zhao J, Zhi Z, Song G, Wang J, Wang C, Ma H, et al. Peroxisome proliferator-activated receptor-Gamma Pro12Ala polymorphism could be a risk factor for gastric cancer. Asian Pac J Cancer Prev. 2015;16:2333–40. 25824760 10.7314/apjcp.2015.16.6.2333

[26] Wang Y, Chen Y, Jiang H, Tang W, Kang M, Liu T, et al. Peroxisome proliferator-activated receptor gamma (PPARG) rs1801282 C>G polymorphism is associated with cancer susceptibility in asians: An updated meta-analysis. Int J Clin Exp Med. 2015;8:12661–73. 26550180 PMC4612865

[27] Xu W, Li Y, Wang X, Chen B, Liu S, Wang Y, et al. PPARgamma polymorphisms and cancer risk: a meta-analysis involving 32,138 subjects. Oncol Rep. 2010;24:579–85. 20596649

[28] Unal E, Aslan EI, Ozturk T, Kurnaz Gomleksiz O, Kucukhuseyin O, Tuzuner MB, et al. Peroxisome proliferator-activated receptor gamma Pro12Ala/C161T genotypes and risky haplotype altering risk of breast cancer: a Turkish case–control study. Biochem Genet. 2021;59:1413–26. 33893920 10.1007/s10528-021-10068-5

[29] Xu X, Xu L, Lang Z, et al. Identification of potential susceptibility loci for non-small cell lung cancer through whole genome sequencing in circadian rhythm genes. Sci Rep. 2025;15:7825. 10.1038/s41598-025-92083-9. 40050692 10.1038/s41598-025-92083-9 PMC11885630

[30] Metwally YF, Elsaid AM, Elsadda RR, Refaat S, Zahran RF. Impact of IL-6 and IL-1β gene variants on non-small-cell lung cancer risk in Egyptian patients. Biochem Genet. 2024;62:3367–88. 38103126 10.1007/s10528-023-10596-2 PMC11427554

[31] Detterbeck FC, Boffa DJ, Kim AW, Tanoue LT. The eighth edition lung cancer stage classification. Chest. 2017;151:193–203. 27780786 10.1016/j.chest.2016.10.010

[32] Metwally YF, Zahran RF, Elsadda RR, Refaat S, Elsaid AM. Association of IL-6 rs1800795 and IL-1 β rs16944 polymorphisms with non-small cell lung cancer in the Egyptian population: a pilot study. Sci J Damietta Fac Sci. 2023;13:8–15.

[33] Elsaid A, Samir eid O, Said SB, Zahran RF. Association of NOS3 (rs 2070744) and SOD2Val16Ala (rs4880) gene polymorphisms with increased risk of ESRD among Egyptian patients. J Genet Eng Biotechnol. 2021;19:158. 34661767 10.1186/s43141-021-00260-w PMC8523625

[34] Elsaid A, Zahran R, Elshazli R, El-Sayed A, Abou Samra M, El-Tarapely F, et al. Genetic polymorphisms of TP53 Arg72Pro and Pro47Ser among Egyptian patients with colorectal carcinoma. Arch Physiol Biochem. 2019;125:255–62. 29560751 10.1080/13813455.2018.1453522

[35] Campa D, Zienolddiny S, Maggini V, Skaug V, Haugen A, Canzian F. Association of a common polymorphism in the cyclooxygenase 2 gene with risk of non-small cell lung cancer. Carcinogenesis. 2004;25:229–35. 14604894 10.1093/carcin/bgh008

[36] Zhang S, Jiang J, Chen Z, Wang Y, Tang W, Chen Y, et al. Relationship of PPARG, PPARGC1A, and PPARGC1B polymorphisms with susceptibility to hepatocellular carcinoma in an eastern Chinese Han population. Onco Targets Ther. 2018;11:4651–60. 30122956 10.2147/OTT.S168274 PMC6087028

[37] Canbay E, Kurnaz O, Canbay B, Bugra D, Cakmakoglu B, Bulut T, et al. PPAR-Gamma Pro12Ala polymorphism and gastric cancer risk in a Turkish population. Asian Pac J Cancer Prev. 2012;13:5875–8. 23317272 10.7314/apjcp.2012.13.11.5875

[38] Chen B, Wang Y, Tang W, Chen Y, Liu C, Kang M, et al. Association between PPARγ, PPARGC1A, and PPARGC1B genetic variants and susceptibility of gastric cancer in an Eastern Chinese population. BMC Med Genomics. 2022;15:274. 36587194 10.1186/s12920-022-01428-0 PMC9805199

[39] Mössner R, Meyer P, Jankowski F, König IR, Krüger U, Kammerer S, et al. Variations in the peroxisome proliferator-activated receptor-γ gene and melanoma risk. Cancer Lett. 2007;246:218–23. 16713673 10.1016/j.canlet.2006.02.022

[40] Sainz J, Rudolph A, Hoffmeister M, Frank B, Brenner H, Chang-Claude J, et al. Effect of type 2 diabetes predisposing genetic variants on colorectal cancer risk. J Clin Endocrinol Metab. 2012;97:E845–51. 22419714 10.1210/jc.2011-2565

[41] Jiang J, Xie Z, Guo JY, Wang Y, Liu C, Zhang S, et al. Association of PPARG rs 1801282 C>G polymorphism with risk of colorectal cancer: From a case-control study to a meta-analysis. Oncotarget. 2017;8:100558–69. 29246001 10.18632/oncotarget.20138 PMC5725043

[42] Wang Y, Cullough MLM, Stevens VL, Rodriguez C, Jacobs EJ, Teras LR, et al. Nested case-control study of energy regulation candidate gene single nucleotide polymorphisms and breast cancer. Anticancer Res. 2007;27:589–93. 17348446

[43] Park B, Shin A, Kim K-Z, Lee Y-S, Hwang J-A, Kim Y, et al. Lack of effects of peroxisome proliferator-activated receptor gamma genetic polymorphisms on breast cancer risk: a case-control study and pooled analysis. Asian Pac J Cancer Prev. 2014;15:9093–9. 25422184 10.7314/apjcp.2014.15.21.9093

[44] Kuruma S, Egawa N, Kurata M, Honda G, Kamisawa T, Ueda J, et al. Case-control study of diabetes-related genetic variants and pancreatic cancer risk in Japan. World J Gastroenterol. 2014;20:17456–62. 25516658 10.3748/wjg.v20.i46.17456 PMC4265605

[45] Qiu H, Wang Y, Kang M, Ding H, Liu C, Tang W, et al. The relationship between IGF2BP2 and PPARG polymorphisms and susceptibility to esophageal squamous-cell carcinomas in the eastern Chinese Han population. Onco Targets Ther. 2017;10:5525–32. 29200867 10.2147/OTT.S145776 PMC5702164

[46] Hasankhani A, Bahrami A, Tavakoli-Far B, Iranshahi S, Ghaemi F, Akbarizadeh MR, et al. The role of peroxisome proliferator-activated receptors in the modulation of hyperinflammation induced by SARS-CoV-2 infection: a perspective for COVID-19 therapy. Front Immunol. 2023;14:1127358. 36875108 10.3389/fimmu.2023.1127358 PMC9981974

[47] Varga T, Czimmerer Z, Nagy L. PPARs are a unique set of fatty acid regulated transcription factors controlling both lipid metabolism and inflammation. Biochim Biophys Acta. 2011;1812:1007–22. 21382489 10.1016/j.bbadis.2011.02.014 PMC3117990

[48] Hernandez-Quiles M, Broekema MF, Kalkhoven E. PPARgamma in metabolism, immunity, and cancer: unified and diverse mechanisms of action. Front Endocrinol. 2021;12:624112. 10.3389/fendo.2021.624112 PMC7953066 33716977

[49] Zhang J, Tang M, Shang J. PPARγ modulators in lung cancer: molecular mechanisms, clinical prospects, and challenges. Biomolecules. 2024;14:190. 38397426 10.3390/biom14020190 PMC10886696

[50] Chi T, Wang M, Wang X, Yang K, Xie F, Liao Z, et al. PPAR-γ modulators as current and potential cancer treatments. Front Oncol. 2021;11:737776. 34631571 10.3389/fonc.2021.737776 PMC8495261

[51] Augimeri G, Giordano C, Gelsomino L, Plastina P, Barone I, Catalano S, et al. The role of pparγ ligands in breast cancer: from basic research to clinical studies. Cancers. 2020;12:2623. 32937951 10.3390/cancers12092623 PMC7564201

[52] Reddy AT, Lakshmi SP, Reddy RC. PPAR γ as a novel therapeutic target in lung cancer. PPAR Res. 2016;2016:8972570. 27698657 10.1155/2016/8972570 PMC5028876

[53] Motawi TK, Shaker OG, Ismail MF, Sayed NH. Peroxisome proliferator-activated receptor gamma in obesity and colorectal cancer: the role of epigenetics. Sci Rep. 2017;7:10714. 28878369 10.1038/s41598-017-11180-6 PMC5587696

[54] Reka AK, Goswami MT, Krishnapuram R, Standiford TJ, Keshamouni VG. Molecular cross-regulation between PPAR-γ and other signaling pathways: implications for lung cancer therapy. Lung Cancer. 2011;72:154–9. 21354647 10.1016/j.lungcan.2011.01.019 PMC3075310

[55] Wang Y, Lei F, Lin Y, Han Y, Yang L, Tan H. Peroxisome proliferator‐activated receptors as therapeutic target for cancer. J Cell Mol Med. 2024;28:e17931. 37700501 10.1111/jcmm.17931 PMC10902584

[56] Angelini S, Ravegnini G, Nannini M, Bermejo JL, Musti M, Pantaleo MA, et al. Folate-related polymorphisms in gastrointestinal stromal tumours: susceptibility and correlation with tumour characteristics and clinical outcome. Eur J Hum Genet. 2015;23:817–23. 25227144 10.1038/ejhg.2014.198 PMC4795063

[57] Wei L, Niu F, Wu J, Chen F, Yang H, Li J, et al. Association study between genetic polymorphisms in folate metabolism and gastric cancer susceptibility in Chinese Han population : a case – control study. Mol Genet Genomic Med. 2019;7:e633. 30884202 10.1002/mgg3.633 PMC6503009

[58] Yoo JY, Kim SY, Hwang JA, Hong SH, Shin A, Choi IJ, et al. Association study between folate pathway gene single nucleotide polymorphisms and gastric cancer in Koreans. Genomics Inform. 2012;10:184–93. 23166529 10.5808/GI.2012.10.3.184 PMC3492654

[59] Stevens VL, Rodriguez C, Sun J, Talbot JT, Thun MJ, Calle EE. No association of single nucleotide polymorphisms in one-carbon metabolism genes with prostate cancer risk. Cancer Epidemiol Biomarkers Prev. 2008;17:3612–4. 19064578 10.1158/1055-9965.EPI-08-0789 PMC2645230

[60] Tong SY, Lee JM, Song ES, Lee KB, Kim MK, Yun YM, et al. The effects of polymorphisms in methylenetetrahydrofolate reductase (MTHFR), methionine synthase (MTR), and methionine synthase reductase (MTRR) on the risk of cervical intraepithelial neoplasia and cervical cancer in Korean women. Cancer Causes Control. 2010;21:23–30. 19760026 10.1007/s10552-009-9430-z

[61] Saad M, Elsaid AM, Mustafa W, El-Khawaga OY. An association of Pro12Ala polymorphism of the PPAR-γ gene with stroke in the Egyptian population. Mansoura J Chem. 2023;61(1):45–51. 10.21608/mjcc.2023.411554.

