# Supplementary material for: Genetic influence of PPAR-γ rs1801282 and MTRR rs162036 variants on non-small cell lung cancer risk in Egyptians
Source: Discov Oncol. 2026 May 7;17:711. doi: 10.1007/s12672-026-04972-8 (PMC13153294; doi:10.1007/s12672-026-04972-8)
Supplement: Supplementary file 1 — Supplementary Material 1. [file 12672_2026_4972_MOESM1_ESM.docx]

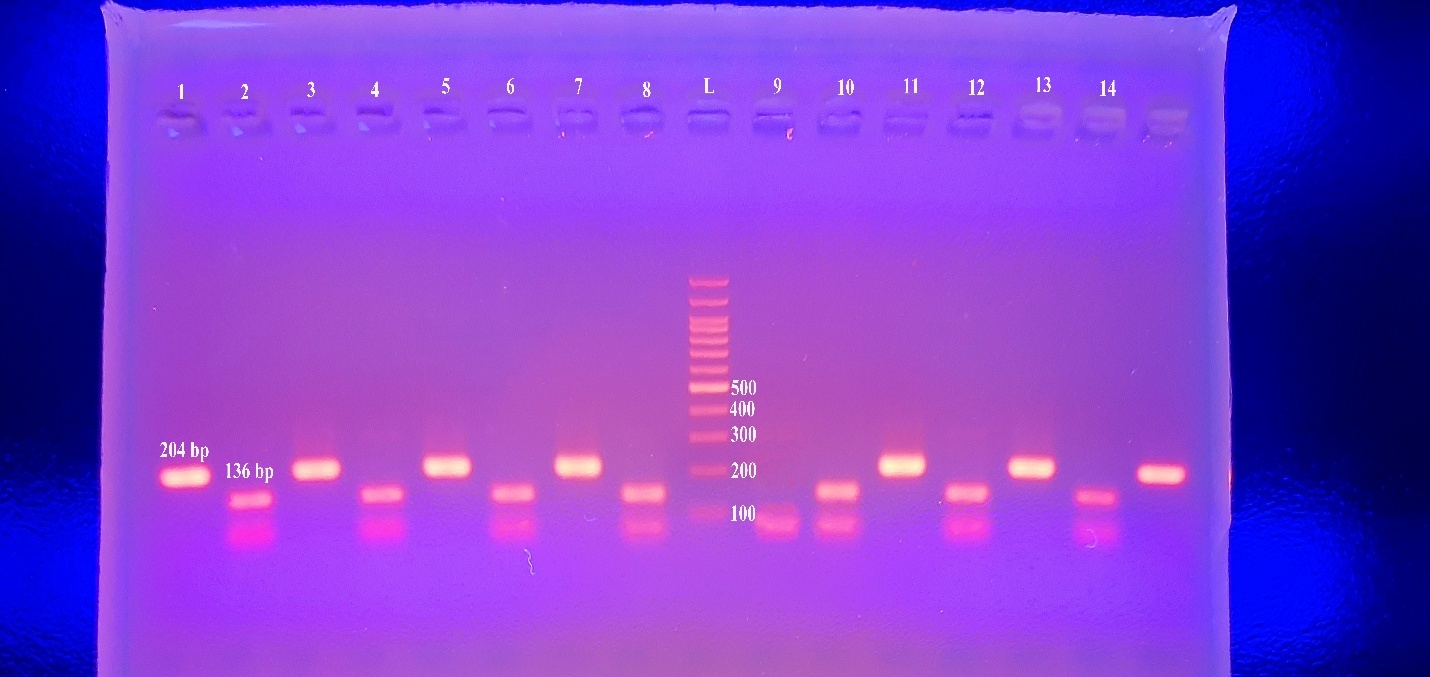


**Fig. S1.** Genotyping products of *MTRR* (rs162036) using ARMS PCR. Lanes (1,2), (5,6), (7,8), (11,12), and (13,14) represent heterozygotes (AG); lanes (3,4) represent rare homozygotes (GG); lanes (9,10) represent common homozygotes (AA). The G-allele is visualized at 204 bp, and the A-allele is visualized at 136 bp. *L* 100 bp DNA ladder. Reactions were performed under optimized PCR conditions to minimize nonspecific amplification.


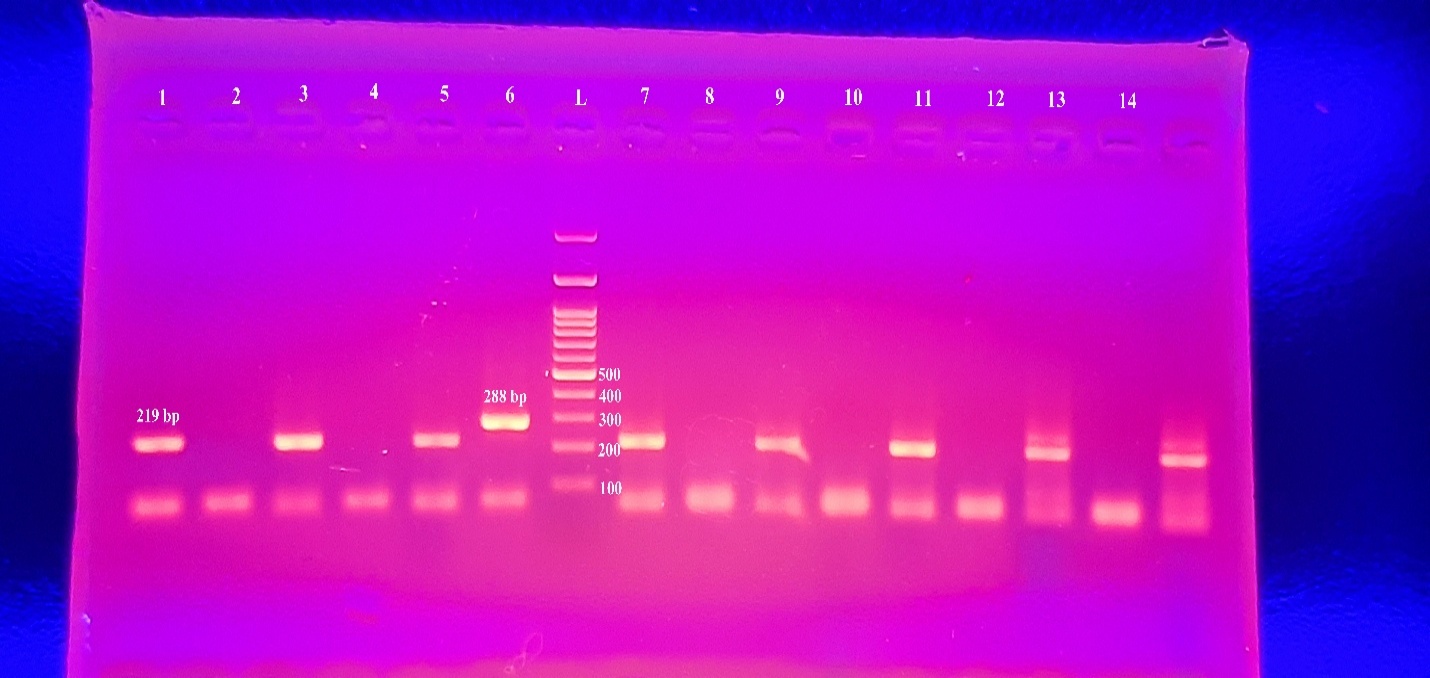


**Fig. S2.** Genotyping products of *PPAR***-γ** (rs1801282) using ARMS PCR. Lanes (1,2), (3,4), (7,8), (9,10), (11,12), and (13,14) represent common homozygotes (CC); lanes (5,6) represent heterozygotes (CG). The C-allele is visualized at 219 bp, and the G-allele is visualized at 288 bp. *L* 100 bp DNA ladder. Reactions were performed under optimized PCR conditions to minimize nonspecific amplification.

**Table S1.** Genotype frequencies of *MTRR* polymorphism stratified by the clinical pathological items of non-small cell lung cancer (NSCLC).

| **Parameter** | | **GG+AG**  **(n=114)** | **AA**  **(n=13)** | ***P* ^a^** | **G allele**  **(n=117)** | **A allele**  **(n=137)** | ***P* ^a^** |
| --- | --- | --- | --- | --- | --- | --- | --- |
| **1. Clinical parameters** |  |  |  |  |  |  |  |
| **Age** | <55/>=55 | 52/62 | 6/7 | 1.0 | 53/64 | 63/74 | 1.0 |
| **Sex** | Female/Male | 45/69 | 6/7 | 0.767 | 45/72 | 57/80 | 0.7 |
| **Smoking** | Smokers/Non | 59/55 | 6/7 | 0.775 | 61/56 | 69/68 | 0.802 |
| **Family history** | Positive/Negative | 13/101 | 1/12 | 1.0 | 14/103 | 14/123 | 0.692 |
| **Surgical history** | Positive/Negative | 39/75 | 6/7 | 0.542 | 41/76 | 49/88 | 1.0 |
| **Medical history** | Positive/Negative | 57/57 | 9/4 | 0.246 | 58/59 | 74/63 | 0.529 |
| **Cough** | Positive/Negative | 44/70 | 5/8 | 1.0 | 46/71 | 52/85 | 0.897 |
| **Dyspnea** | Positive/Negative | 40/74 | 4/9 | 0.775 | 41/76 | 47/90 | 1.0 |
| **Chest pain** | Positive/Negative | 34/80 | 6/7 | 0.344 | 34/83 | 46/91 | 0.499 |
| **Hemoptysis** | Positive/Negative | 8/106 | 1/12 | 1.0 | 8/109 | 10/127 | 1.0 |
| **Tumor size (T)** | T1, T2 | 11 | 1 | 1.0 | 11 | 13 | 1.0 |
|  | T3, T4 | 103 | 12 |  | 106 | 124 |  |
| **Lymph node (N)** | N0, N1 | 28 | 3 | 1.0 | 29 | 33 | 1.0 |
|  | N2, N3 | 86 | 10 |  | 88 | 104 |  |
| **Stage** | Stage 1, 2 | 5 | 0 | 0.659 | 5 | 5 | 1.0 |
|  | Stage 3, 4 | 109 | 13 |  | 112 | 132 |  |
| **Grade** | Mild | 3 | 0 | 1.0 | 3 | 3 | 1.0 |
|  | Moderate, High | 111 | 13 |  | 114 | 134 |  |
| **Histological types** | Adenocarcinoma | 89 | 10 | 1.0 | 91 | 107 | 1.0 |
|  | Squamous cell carcinoma carcinoma rcinoma carcinoma | 12 | 1 |  | 12 | 14 |  |
|  | Large cell carcinoma | 13 | 2 |  | 14 | 16 |  |
| **2. Biochemical parameters** |  |  |  |  |  |  |  |
| PT |  | 13.0±0.11 | 13.7±0.4 | 0.062 | 12.9±0.1 | 13.1±0.1 | 0.347 |
| INR, M ± SE |  | 1.08±0.01 | 1.12±0.03 | 0.275 | 1.08±0.01 | 1.09±0.01 | 0.576 |
| ALT (U/L), M ± SE |  | 24.5±1.3 | 16.7±1.4 | 0.052 | 24.6±1.3 | 22.9±1.1 | 0.323 |
| AST (U/L), M ± SE |  | 23.9±1.1 | 19.1±1.2 | 0.153 | 24.0±1.09 | 23.0±0.96 | 0.487 |
| Total bilirubin (mg/dL), M ± SE |  | 0.55±0.2 | 0.54±0.07 | 0.892 | 0.55±0.01 | 0.55±0.01 | 0.958 |
| Creatinine (mg/dL), M ± SE |  | 0.89±0.01 | 0.87±0.09 | 0.727 | 0.89±0.01 | 0.88±0.01 | 0.825 |
| Hemoglobin (g/dL), M ± SE |  | 12.7±0.17 | 12.9±0.48 | 0.767 | 12.7±0.16 | 12.8±0.15 | 0.697 |
| WBCs count (× 10^9^/L), M ± SE |  | 10.4±0.46 | 9.5±0.62 | 0.517 | 10.6±0.49 | 10.1±0.36 | 0.413 |
| Platelet count (× 10^9^/L), M ± SE |  | 309.8±8.9 | 278.7±22.6 | 0.264 | 310.9±8.7 | 303±8.0 | 0.507 |
| NEUT (%), M ± SE |  | 65.6±1.09 | 63.4±2.6 | 0.511 | 65.7±1.08 | 65.1±0.9 | 0.682 |
| LYMPH (%), M ± SE |  | 22.5±0.97 | 25.6±3.1 | 0.323 | 22.4±0.96 | 23.2±0.89 | 0.564 |
| **3. Tumor markers** |  |  |  |  |  |  |  |
| CEA (ng/mL) | Positive/Negative | 72/42 | 8/5 | 1.0 ^b^ | 74/43 | 86/51 | 1.0 |
| EGFR | Mutant/Wild | 29/85 | 2/11 | 0.519 ^b^ | 31/86 | 31/106 | 0.558 |

**^a^** t test for quantitative data and Fisher’s exact test for qualitative data, *p* > 0.05: non-significant.*CEA* carcinoembryonic antigen, *EGFR* epidermal growth factor receptor

**Table S2.** Genotype frequencies of *PPAR-γ* polymorphism stratified by the clinical pathological items of non-small cell lung cancer (NSCLC).

| **Parameter** | | **CG**  **(n=23)** | **CC**  **(n=104)** | ***P*** | **G allele**  **(n=23)** | **C allele**  **(n=104)** | ***P* ^a^** |
| --- | --- | --- | --- | --- | --- | --- | --- |
| **1. Clinical parameters** |  |  |  |  |  |  |  |
| **Age** | <55/>=55 | 8/15 | 50/54 | 0.35 | 8/15 | 108/123 | 0.38 |
| **Sex** | Female/Male | 10/13 | 41/63 | 0.81 | 10/13 | 92/139 | 0.82 |
| **Smoking** | Smokers/Non | 11/12 | 54/50 | 0.82 | 11/12 | 119/112 | 0.82 |
| **Family history** | Positive/Negative | 1/22 | 13/91 | 0.46 | 1/22 | 27/204 | 0.48 |
| **Surgical history** | Positive/Negative | 6/17 | 39/65 | 0.34 | 6/17 | 84/147 | 0.37 |
| **Medical history** | Positive/Negative | 13/10 | 53/51 | 0.65 | 13/10 | 119/112 | 0.66 |
| **Cough** | Positive/Negative | 7/16 | 42/62 | 0.48 | 7/16 | 91/140 | 0.50 |
| **Dyspnea** | Positive/Negative | 8/15 | 36/68 | 1.0 | 8/15 | 80/151 | 1.0 |
| **Chest pain** | Positive/Negative | 8/15 | 32/72 | 0.80 | 8/15 | 72/159 | 0.81 |
| **Hemoptysis** | Positive/Negative | 0/23 | 9/95 | 0.36 | 0/23 | 18/213 | 0.38 |
| **Tumor size (T)** | T1, T2 | 3 | 9 | 0.45 | 3 | 21 | 0.46 |
|  | T3, T4 | 20 | 95 |  | 20 | 210 |  |
| **Lymph node (N)** | N0, N1 | 9 | 22 | 0.10 | 9 | 53 | 0.12 |
|  | N2, N3 | 14 | 82 |  | 14 | 178 |  |
| **Stage** | Stage 1, 2 | 0 | 5 | 0.58 | 0 | 10 | 0.60 |
|  | Stage 3, 4 | 23 | 99 |  | 23 | 221 |  |
| **Grade** | Mild | 1 | 2 | 0.45 | 1 | 5 | 0.43 |
|  | Moderate, High | 22 | 102 |  | 22 | 226 |  |
| **Histological types** | Adenocarcinoma | 20 | 79 | 0.55 | 20 | 178 | 0.58 |
|  | Squamous cell carcinoma | 2 | 11 |  | 2 | 24 |  |
|  | Large cell carcinoma | 1 | 14 |  | 1 | 29 |  |
| **2. Biochemical parameters** |  |  |  |  |  |  |  |
| PT |  | 13.1±0.24 | 13.1±0.14 | 0.905 | 13.1±0.24 | 13.1±0.09 | 0.91 |
| INR, M ± SE |  | 1.08 ± 0.02 | 1.09 ± 0.01 | 0.76 | 1.09 ± 0.02 | 1.09 ±0.0 | 0.77 |
| ALT (U/L), M ± SE |  | 27.1 ± 4.2 | 23.1 ± 1.2 | 0.24 | 27.1 ± 4.2 | 23.4 ± 0.89 | 0.26 |
| AST (U/L), M ± SE |  | 25.4 ± 3.3 | 24.1 ± 1.5 | 0.71 | 25.4 ± 3.3 | 24.2 ± 1.03 | 0.73 |
| Total bilirubin (mg/dL), M ± SE |  | 0.53 ± 0.05 | 0.56 ± 0.02 | 0.65 | 0.53 ± 0.05 | 0.56 ± 0.01 | 0.67 |
| Creatinine (mg/dL), M ± SE |  | 0.96 ± 0.04 | 0.85 ± 0.02 | 0.06 | 0.96 ± 0.04 | 0.86 ± 0.01 | 0.07 |
| Hemoglobin (g/dL), M ± SE |  | 13.0 ± 0.37 | 12.7 ± 0.18 | 0.46 | 13.0 ± 0.37 | 12.8 ± 0.12 | 0.48 |
| WBCs count (× 10^9^/L), M ± SE |  | 9.6 ± 0.79 | 10.5 ± 0.51 | 0.39 | 9.6 ± 0.79 | 10.4 ± 0.33 | 0.41 |
| Platelet count (× 10^9^/L), M ± SE |  | 292 ± 18.1 | 308 ± 9.8 | 0.48 | 292 ± 18.1 | 306 ± 6.5 | 0.50 |
| NEUT (%), M ± SE |  | 65.8 ± 2.3 | 66.3 ± 1.1 | 0.84 | 65.8 ± 2.3 | 66.3 ± 0.77 | 0.85 |
| LYMPH (%), M ± SE |  | 22.6 ± 1.9 | 22.6 ± 1.06 | 0.97 | 22.6 ± 1.9 | 22.6 ± 0.7 | 0.97 |
| **3. Tumor markers** |  |  |  |  |  |  |  |
| CEA (ng/mL) | Positive/Negative | 16/7 | 64/40 | 0.63 | 16/7 | 144/87 | 0.65 |
| EGFR | Mutant/Wild | 5/18 | 26/78 | 1.0 | 5/18 | 57/174 | 1.0 |

**^a^** t test for quantitative data and Fisher’s exact test for qualitative data, *p* > 0.05: non-significant.

*CEA* carcinoembryonic antigen, *EGFR* epidermal growth factor receptor

**Table S3.** Worldwide distribution of the *PPAR-γ* (rs1801282) polymorphisms among cases of different types of cancers and controls recruited between 2004 and 2025

|  | | | | | | | | Cases, n(%) | | | | | | | | Controls, n(%) | | | | | | | | Dominant | | | |  | | Notes/Issues |  |  |  |  |  |
| --- | --- | --- | --- | --- | --- | --- | --- | --- | --- | --- | --- | --- | --- | --- | --- | --- | --- | --- | --- | --- | --- | --- | --- | --- | --- | --- | --- | --- | --- | --- | --- | --- | --- | --- | --- |
| Author | | | | **Year** | | **Ethnicity** | | **Cancer Type** | | **N** | | **CC** | | **CG** | | **GG** | | **N** | | **CC** | | **CG** | | **GG** | | **OR (95% CI)** | | **P**  **value** | |  | | Confirmed | | |  |
| This study | | | | 2025 | | Egyptian | | Non-small Cell Lung Cancer | | 127 | | 104 (81.9%) | | 23 (18.1%) | | 0 (0%) | | 138 | | 133 (96.4%) | | 5 (3.6%) | | 0 (0%) | | 5.88 (2.08–20.36) | | **0.0001** | |  | | Confirmed | | |  |
| Chen B | | | | | 2022 | | Eastern Chinese | | Gastric Cancer | | 490 | | 452 (92.2%) | | 34 (6.9%) | | 1 (0.2%) | | 1476 | | 1317 (89.2%) | | 151 (10.2%) | | 4 (0.27%) | | 0.66 (0.43–0.97) | | **0.0337** | |  | | Confirmed | | |
| Unal E | | | | | 2021 | | Turkish | | Breast Cancer | | 95 | | 91 (95.8%) | | 2 (2.1%) | | 2 (2.1%) | | 119 | | 95 (79.8%) | | 23 (19.3%) | | 1 (0.8%) | | 0.17 (0.04–0.54) | | **0.0004** | |  | | Confirmed | | |
| Zhang S | | | | | 2018 | | Eastern Chinese | | Hepatocellular Cancer | | 584 | | 542 (94.3%) | | 30 (5.22%) | | 3 (0.52%) | | 923 | | 823 (89.4%) | | 95 (10.3%) | | 3 (0.33%) | | 0.51 (0.33–0.78) | | **0.001** | |  | | Confirmed | | |
| Jiang J | | | | | 2017 | | Eastern Chinese | | Colorectal Cancer | | 387 | | 357 (93.2%) | | 25(6.5%) | | 1 (0.26%) | | 1536 | | 1384 (90.2%) | | 144 (9.4%) | | 5 (0.33%) | | 0.68 (0.42–1.05) | | 0.0912 | |  | | Confirmed | | |
| Qiu H | | | | | 2017 | | Eastern Chinese | | Esophageal Squamous-cell Cancer | | 507 | | 440 (87.3%) | | 63 (12.5%) | | 1 (0.2%) | | 1496 | | 1334 (89.6%) | | 151 (10.1%) | | 4 (0.27%) | | 1.25 (0.90–1.72) | | 0.1616 | |  | | Confirmed | | |
| Kuruma S | | | | | 2014 | | Japanese | | Pancreatic Cancer | | 360 | | 334 (92.7%) | | 26 (7.2%) | | 0 (0.0%) | | 400 | | 373 (93.3%) | | 27 (6.7%) | | 0 (0.0%) | | 1.08 (0.59-1.96) | | 0.8868 | |  | | Confirmed | | |
| Park B | | | | | 2014 | | korean | | Breast Cancer | | 456 | | 413 (90.8%) | | 40 (8.8%) | | 2 (0.4%) | | 461 | | 412 (90.6%) | | 42 (9.2%) | | 1 (0.2%) | | 0.97 (0.61-1.56) | | 1.0 | |  | | Confirmed | | |
| Sainz J | | | | | 2012 | | German | | Colorectal Cancer | | 1798 | | 1354 (74.8%) | | 415 (22.9%) | | 32 (1.8%) | | 1810 | | 1334 (74.2%) | | 427 (23.7%) | | 22 (1.2%) | | 0.98 (0.84-1.14) | | 0.817 | |  | | Confirmed | | |
| Canbay E | | | | | 2012 | | Turkish | | Gastric Cancer | | 86 | | 68 (79.0%) | | 14 (16.3%) | | 4 (4.7%) | | 129 | | 116 (89.9%) | | 12 (9.3%) | | 1 (0.77%) | | 2.36 (1.02-5.58) | | **0.0305** | |  | | Confirmed | | |
| Wang Y | | 2007 | | American | | Breast Cancer | | 488 | | 376 (78.7%) | | 87 (18.2%) | | 15 (3.1%) | | 488 | | 375 (78.5%) | | 98 (20.5%) | | 5 (1.0%) | | 0.99 (0.72-1.36) | | 1.0 | |  | | Confirmed | | |  |  |  |
| Mössner R | | | | 2007 | | German | | Melanoma | | 335 | | 239 (71.6%) | | 84 (25.1%) | | 11 (3.3%) | | 355 | | 258 (73.5%) | | 86 (24.5%) | | 7 (2.0%) | | 1.10 (0.78-1.56) | | 0.6075 | |  | | Confirmed | | |  |
| Campa D | | | | 2004 | | Norwegian | | Non-small Cell Lung Cancer | | 246 | | 2 (0.8%) | | 52 (21.1%) | | 192 (78%) | | 212 | | 4 (1.8%) | | 47 (22.2%) | | 161 (76%) | | 0.97 (0.61-1.56) | | 0.4222 | |  | | Confirmed | | |  |

**Table S4.** Worldwide distribution of the *MTRR* rs162036 polymorphism among cases of different types of cancers and controls recruited between 2010 and 2025

|  | | | | Cases, n(%) | | | | Controls, n(%) | | | | Dominant | |
| --- | --- | --- | --- | --- | --- | --- | --- | --- | --- | --- | --- | --- | --- |
| Author | **Year** | **Ethnicity** | **Cancer Type** | **N** | **AA** | **AG** | **GG** | **N** | **AA** | **AG** | **GG** | **OR (95% CI)** | **P value** |
| This study | 2025 | Egyptian | Non-small Cell Lung Cancer | 127 | 13 (10.2) | 111 (87.4) | 3 (2.4) | 138 | 8 (5.8) | 128 (92.8) | 2 (1.4) | 0.54 (0.19–1.47) | 0.255 |
| Wei L | 2019 | Chinese Han | Gastric Cancer | 681 | 477 (70) | 187 (27.5) | 17 (2.5) | 756 | 519 (68.7) | 210 (27.8) | 26 (3.4) | 0.94 (0.75–1.19) | 0.606 |
| Luo WP | 2016 | Chinese | Breast Cancer | 576 | 360 (63.2) | 199 (34.9) | 11 (1.9) | 576 | 371 (64.4) | 174 (30.2) | 31 (5.4) | 1.06 (0.82–1.35) | 0.667 |
| Angelini S | 2015 | Caucasian | Gastrointestinal Stromal Tumours | 60 | 48 (80) | 11 (18.3) | 1 (1.7) | 153 | 112 (73.7) | 38 (25) | 2 (1.3) | 0.70 (0.31–1.45) | 0.379 |
| Yoo JY | 2012 | Koreans | Gastric Cancer | 1246 | 837 (67.2) | 373 (29.9) | 36 (2.9) | 368 | 258 (70.1) | 98 (26.6) | 12 (3.3) | 1.15 (0.88–1.49) | 0.309 |
| Pardini BK | 2011 | Czechs | Colorectal Cancer | 666 | 495 (83.9) | 90 (15.3) | 5 (0.8) | 1377 | 1044 (80.7) | 231 (17.9) | 18 (1.4) | 0.80 (0.61–1.05) | 0.108 |
| Tong SY | 2010 | Korean | Cervical Cancer | 155 | 102 (70) | 44 (30) | | 440 | 284 (67) | 143 (33) | | 0.86 (0.56–1.31) | 0.476 |
